# A phase 1 clinical trial of SP16, a first-in-class anti-inflammatory LRP1 agonist, in healthy volunteers

**DOI:** 10.1371/journal.pone.0247357

**Published:** 2021-05-06

**Authors:** George F. Wohlford, Leo F. Buckley, Dinesh Kadariya, Taeshik Park, Juan Guido Chiabrando, Salvatore Carbone, Virginia Mihalick, Matthew S. Halquist, Adam Pearcy, Dana Austin, Cohava Gelber, Antonio Abbate, Benjamin Van Tassell

**Affiliations:** 1 Virginia Commonwealth University School of Pharmacy, Virginia Commonwealth University, Richmond, Virginia, United States of America; 2 Pauley Heart Center, Virginia Commonwealth University, Richmond, Virginia, United States of America; 3 Serpin Pharma LLC, Manassas, Virginia, United States of America; Campus Bio-Medico University of Rome, ITALY

## Abstract

**Background:**

Endogenous serine protease inhibitors are associated with anti-inflammatory and pro-survival signaling mediated via Low-density lipoprotein receptor-related protein 1 (LRP1) signaling. SP16 is a short polypeptide that mimics the LRP1 binding portion of alpha-1 antitrypsin.

**Methods:**

A pilot phase I, first-in-man, randomized, double blind, placebo-controlled safety study was conducted to evaluate a subcutaneous injection at three dose levels of SP16 (0.0125, 0.05, and 0.2 mg/kg [up to 12 mg]) or matching placebo in 3:1 ratio in healthy individuals. Safety monitoring included vital signs, laboratory examinations (including hematology, coagulation, platelet function, chemistry, myocardial toxicity) and electrocardiography (to measure effect on PR, QRS, and QTc).

**Results:**

Treatment with SP16 was not associated with treatment related serious adverse events. SP16 was associated with mild-moderate pain at the time of injection that was significantly higher than placebo on a 0–10 pain scale (6.0+/-1.4 [0.2 mg/kg] versus 1.5+/-2.1 [placebo], P = 0.0088). No differences in vital signs, laboratory examinations and electrocardiography were found in those treated with SP16 versus placebo.

**Conclusion:**

A one-time treatment with SP16 for doses up to 0.2 mg/kg or 12 mg was safe in healthy volunteers.

## Introduction

Low-density lipoprotein receptor-related protein 1 (LRP1) is a scavenger and regulatory receptor expressed ubiquitously on the surface of cellular membranes in humans [1–3]. The binding of LRP1 by protease-inhibitor complexes results in phosphorylation of the protein kinase Akt pathway that is associated with multiple pro-survival and anti-inflammatory pathways. It is hypothesized that if these pathways are activated in a timely manner, they may provide benefits in preventing injury related to ischemia [4–6].

**Fig 1:**
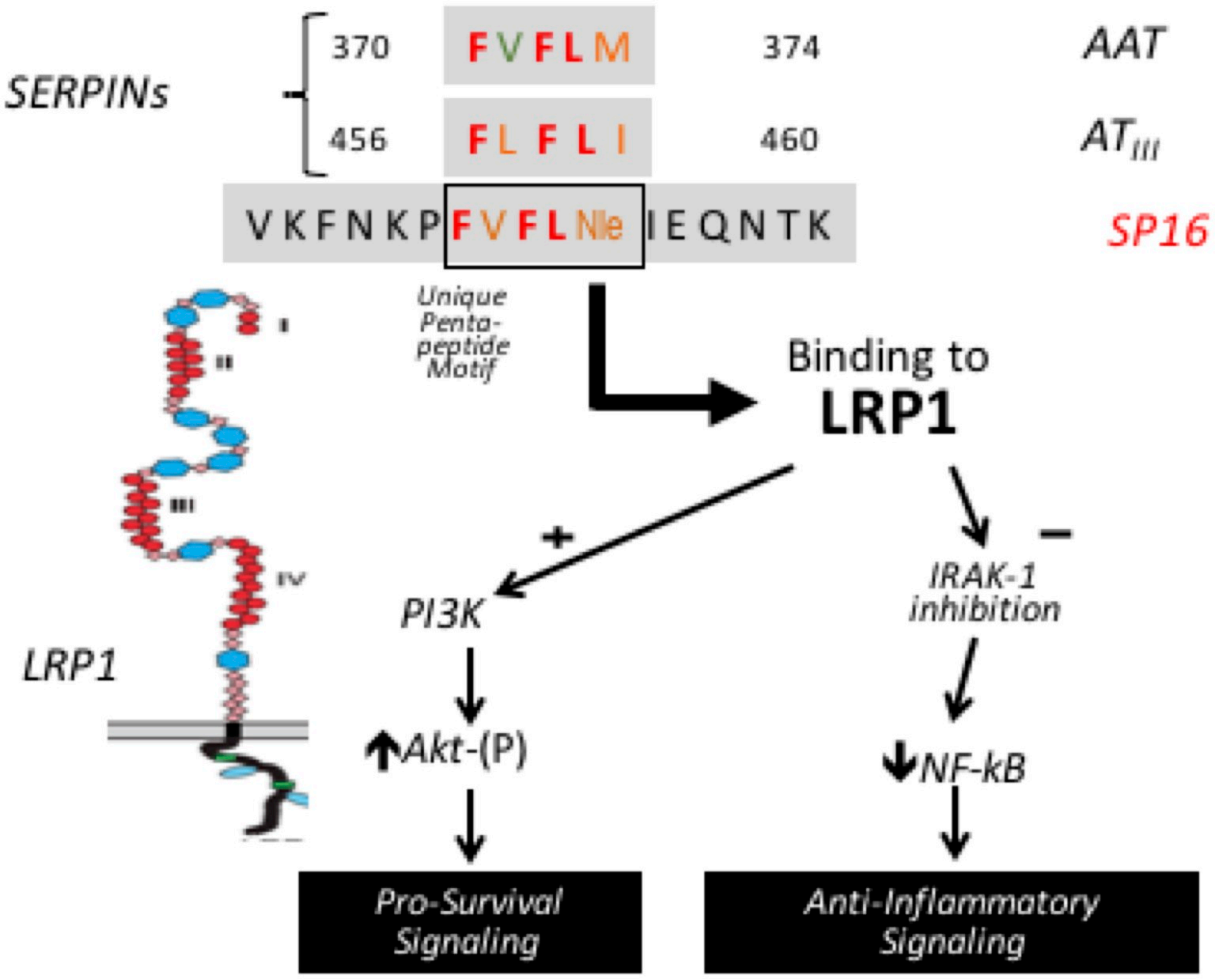
SP16, a novel, synthetic LRP1 agonist.

Serine protease inhibitors, known as SERPINs, are endogenous proteins that inactivate proteolytic enzymes released during inflammatory responses and have been found to transduce intracellular signals through actions on LRP1 [2, 5, 6]. SP16 is a short amino acid sequence designed to mimic the LRP1 binding portion of the endogenous SERPINs like alpha-1 antitrypsin (AAT) (Fig 1). *In-vitro* studies have shown the sequence to have a high binding affinity for the LRP1 receptor [5]. AAT has been associated with infusion-related mild-moderate headache, musculoskeletal discomfort and sore throat as well as asymptomatic elevations in aminotransferase levels less than 5 times the upper limit of normal that resolved within 3-months. SP16 lacks cross-reactivity with more than 100 kinases and receptors in vitro and anti-SP16 antibodies have not been detected in animals. There is also a theoretical pharmacodynamic interaction between SP16 and unfractionated heparin or low molecular weight heparin due to the serine protease inhibiting effects of antithrombin, which facilitates the anticoagulant effects of heparinoids [7]. This potential interaction has added significance given the targeted use of SP16 in patients with acute myocardial infarction.

In the murine model, SP16 administered after a simulated myocardial infarction was shown to reduce the inflammatory response and was found to have infarct-sparing effects on heart tissue [5]. The present work describes a first-in-human, phase I, study designed to evaluate the safety, tolerability, and pharmacokinetics (PK) and pharmacodynamics (PD) of a single, escalating subcutaneous doses of SP16 in healthy volunteers.

## Methods

The study protocol was approved by the Western Internal Review Board (Puyallup, WA) on July 11^th^, 2018. All patients provided informed consent prior to undergoing any study procedures. All study procedures were conducted in the Clinical Research Unit at Virginia Commonwealth University (VCU) in Richmond, Virginia. The trial was registered with clinicaltrials.gov (NCT03651089) under an IND held by the sponsor. The authors confirm that all ongoing and related trials for this drug/intervention are registered.

### Study design

**Fig 2:**
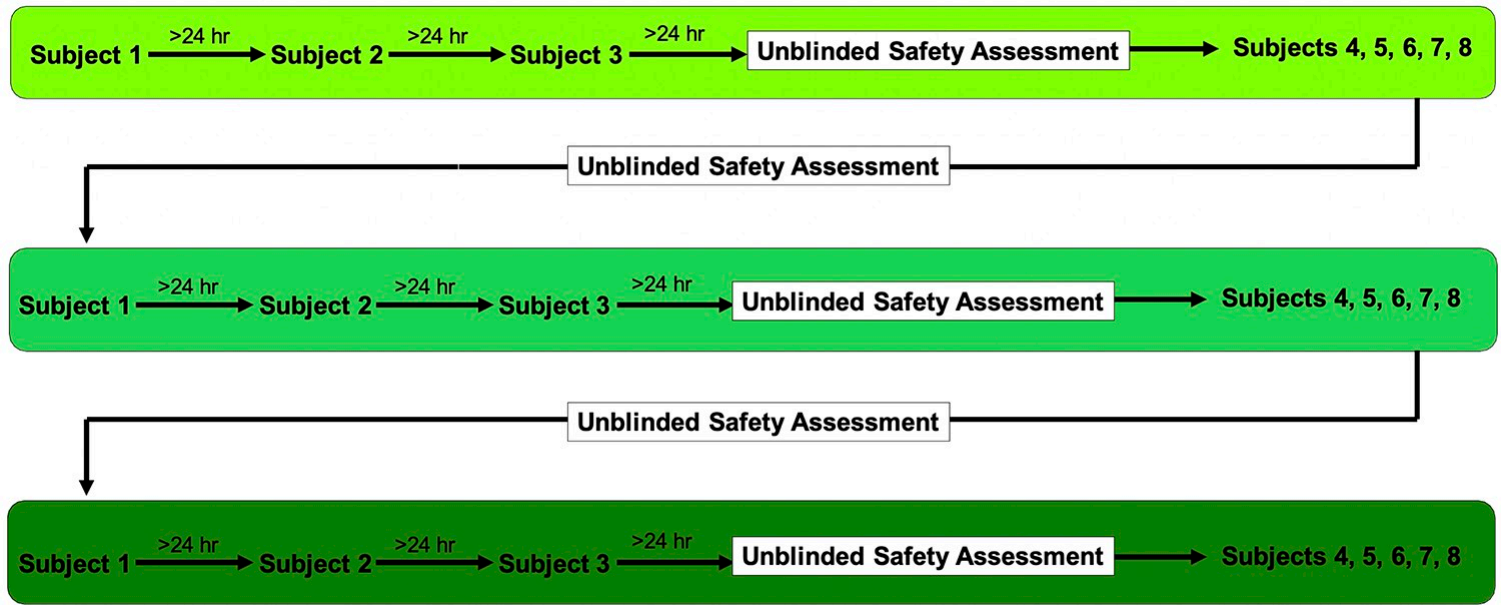
Schematic of the study design.

A pilot phase I, first-in-man, randomized, double blind, placebo-controlled safety study was conducted to evaluate a one-time subcutaneous dose of SP16 or matching placebo in healthy individuals. Three separate dose cohorts utilizing sequentially increasing dose levels (0.0125, 0.05, 0.2 mg/kg) were recruited and randomized in a 3:1 allocation ratio to receive a single administration of SP16 or matching placebo (Fig 2). Doses of SP16 were capped at a maximum dose of 12 mg. Patients requiring a volume greater than 2 mL had the dose split into two equal volume injections that were sequentially administered separately on opposite sides of the abdomen. For the first 3 patients in each cohort, each dose administration was separated by a 24-hour observation period for sentinel adverse drug reaction monitoring. Following the observation period of the third patient in each cohort, a data safety review and adverse event assessment was completed before dosing the remaining patients in the cohort. A total of 6 patients received placebo (2 from each cohort) and together created a control cohort for comparison of safety parameters.

### Dose selection

Dose selections were made in accordance to United States Food & Drug Administration (FDA) Guidance for estimating the maximum safe starting dose and based on data obtained in rat and dog animal models. In rats, doses of SP16 60 mg/kg were administered without any observed toxicity. When the FDA human equivalent dose conversion factor of 6.2 and FDA safety factor of 10 were applied, it was estimated a dose of 0.97 mg/kg would have acceptable safety. The dose for the first cohort exceeded the FDA standards with a calculated safety factor of 77.4. In a phase 2 trial of plasma derived AAT administered at a dose of 60 mg/kg in patients with acute myocardial infarction, the treatment was well tolerated and free of treatment related adverse events. It is expected that SP16’s specificity for LRP1 will provide a greater safety margin than plasma-derived AAT.

### Study medication and randomization

The peptide was manufactured using a solid-phase peptide synthesis resin procedure for the production of crude peptide. Salt exchange and lyophilization techniques were used to purify the peptide to be used for injection (Chinese peptide company, Inc). The peptide used for injection was formulated in double distilled water titrated to a pH of 5.9 for a final drug concentration of 3 mg/mL and stored frozen at -20°C in amber vials.

Each dose cohort of 8 patients was randomized (3:1) to SP16 or placebo such that 6 patients within each dose cohort received SP16 and 2 patients received placebo. The placebo patients within each cohort were then pooled together such that 6 patients received each dose of SP16 and 6 patients received placebo. Randomization was performed by the local investigational drug services using a dedicated online randomization algorithm. The investigational pharmacy was notified upon patient arrival for a drug administration visit. Based on subject allocation, SP16 or placebo vials were thawed and prepared in a blinded fashion. Syringes were stored at 4°C until the time of the injection. Subject treatment allocation was concealed from the investigators until completion of all appropriate data collection (pre-specified safety analyses and final study completion).

### Inclusion and exclusion criteria

In order to be eligible to participate, subjects needed to be between the ages of 18 to 59, willing to comply with study procedures, and currently be using highly effective contraception. Patients were excluded for a history of an acute or chronic illness requiring medications, with a febrile illness within the past 14 days, a known allergic reactions to components of the study agent, received treatment with another investigational drug within the prior 30 days, tobacco use within the prior 60 days, with household contacts who are immunocompromised, with a chronic infection, malignancy (of any kind), a substance abuse disorder, or pregnancy or breastfeeding.

### Blood sampling and monitoring procedures

Subjects were admitted to a clinical research unit for 12 hours and an intravenous cannula for blood sampling was placed. Blood sampling occurred prior to the dose, at 15, 30, 45, 60, 90, 120, 180 minutes and then at 6 and 12 hours into two 4mL ethylenediaminetetraacetic acid anticoagulated vacutainers at each timepoint and centrifuged (10 mins, 1500g, 4°C). The resulting plasma was immediately aliquoted into cryoresistant vials and stored at -80°C for batch analysis pending assay development.

Vital signs were measured prior to treatment and monitored just prior to each pharmacokinetic blood draw. A complete history and physical exam were completed before and 12 hours after the treatment. A comprehensive metabolic panel, complete blood cell count and markers of cardiac injury drawn at baseline and 12 hours. Markers of cardiac injury were also drawn 6 hours from the time of drug administration. A 12-lead electrocardiogram (ECG) was obtained at baseline, 30 minutes, and 12 hours after drug administration. QT intervals were corrected using the Fridericia equation. Measures of hemostasis and platelet function tests (including assessment of P2Y12 reactivity by VerifyNow^®^ assay) were obtained prior to the dose, 60 minutes and at 6 hours. Injection site pain was assessed using an 11-point scale (range from 0 to 10) immediately following the injection and again at 30 minutes, 12 hours, 48–72 hours, and 7 days. Adverse events were assessed at each visit and repeat safety labs were drawn at the 48–72 hours visit. A complete safety parameter testing schedule can be found in the appendix (S1 Table in S1 File).

### Statistical methods

Baseline measurements and demographic characteristics are summarized with medians and interquartile range and the Kruskal-Wallis test was used for between group comparisons. Descriptive summaries of categorical measurements consist of frequencies and proportions and the chi-square test used for between group comparisons.

Drug effects on safety parameters were evaluated by repeated measures analysis of variance (ANOVA) with testing for time group interactions. Pain with injection was compared using a one-way ANOVA followed by Tukey’s post-hoc analysis for within group comparisons. An alpha level of 5% was set to determine statistical significance for statistical tests. All analyses were conducted with SPSS Statistics Version 27 (Cary, North Carolina, United States of America).

## Results

**Fig 3:**
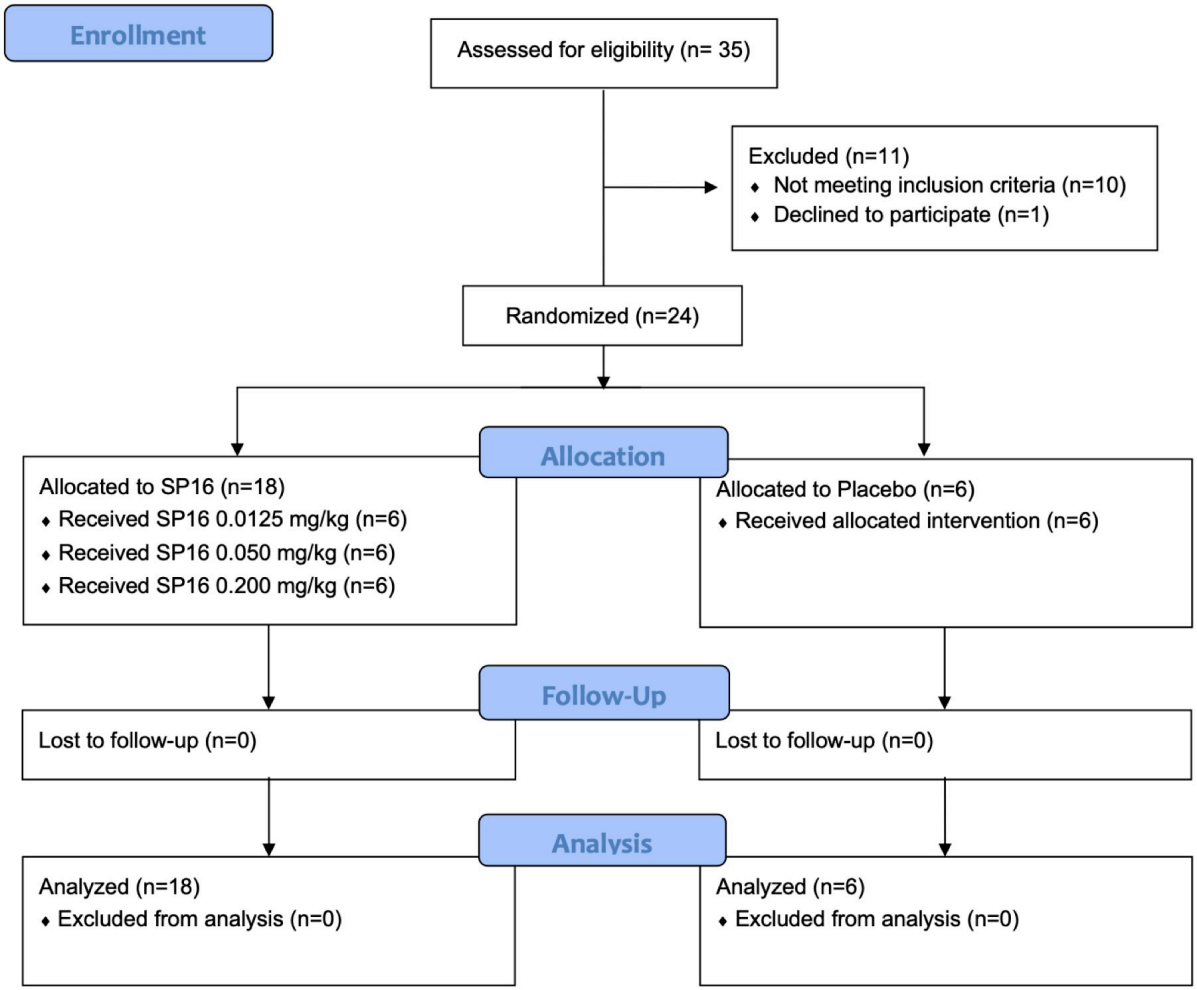
CONSORT participant flow diagram.

The first subject was dosed on August 1^st^, 2018 and the final patient visit was completed by January 15^th^, 2019. A total of 35 subjects were screened and 25 healthy volunteers were enrolled (Fig 3). There were no differences in baseline demographic characteristics (Table 1). Study enrollment proceeded from dose level through dose level 3 without meeting safety criteria for stopping.

**Table 1:** Baseline demographics.

	Placebo	SP16 0.0125 mg/g	SP 16 0.050 mg/kg	SP16 0.200 mg/kg	P-value
Number of subjects	6	6	6	6	N/A
Age, years (IQR)	37 (21, 48)	21 (21, 32)	35 (25,42)	23 (20, 27)	0.07
Female (%)	4 (67%)	3 (50%)	2 (33%)	2 (33%)	0.60
non-Hispanic, n (%)	6 (100%)	6 (100%)	6 (100%)	6 (100%)	1
Caucasian, n (%)	3 (50%)	3 (50%)	5 (83%)	5 (83%)	0.51
Asian, n (%)	1 (17%)	2 (33%)	1 (17%)	1 (17%)	
Black, n (%)	2 (33%	1 (17%)	0 (0%)	0 (0%)	
Height, cm (IQR)	167 (161, 175)	168 (162, 177)	170 (163, 182)	171 (166, 182)	0.75
Weight, kg (IQR)	76 (68, 92)	69 (58, 71)	65 (58, 81)	73 (64, 98)	0.27
HR, bpm (IQR)	78 (71, 84)	65 (53, 81)	69 (53, 79)	76 (67, 87)	0.29
Systolic BP, mmHg (IQR)	121 (106, 130)	123 (105, 127)	115 (105, 127)	124 (117, 133)	0.69
Diastolic BP. mmHg (IQR)	74 (65, 82)	76 (68, 83)	70 (65, 80)	77 (74, 85)	0.59

BP = blood pressure, bpm = beats per minute, cm = centimeter, kg = kilogram, IQR = interquartile range n = Number, mmHg = millimeter of mercury

SP16 was found to have no effects on clinical biomarkers (S1 Table in S1 File). In placebo patients, the median IQR for aspartate aminotransferase (AST) levels at baseline, 12- and 48/72-hours were 23 (21, 30) units/L, 22.5 (21, 29) units/L and 24 (22, 27) units/L, respectively. In the 0.0125 mg/kg SP16 group AST levels were 29.5 (17, 48.5) units/L, 29.5 (22, 44.5) units/L and 30 (16, 43.5), respectively. For the 0.050 mg/kg SP16 cohort the AST levels were 22 (18, 25) units/L, 24 (16, 26) units/L, and 22 (17, 28) units/L, respectively. And for the 12 mg (0.200 mg/kg) SP16 cohort, the AST levels were 27 (17, 32) units/L, 22 (18, 29) units/L, and 22 (19, 28) units/L, respectively.

**Fig 4:**
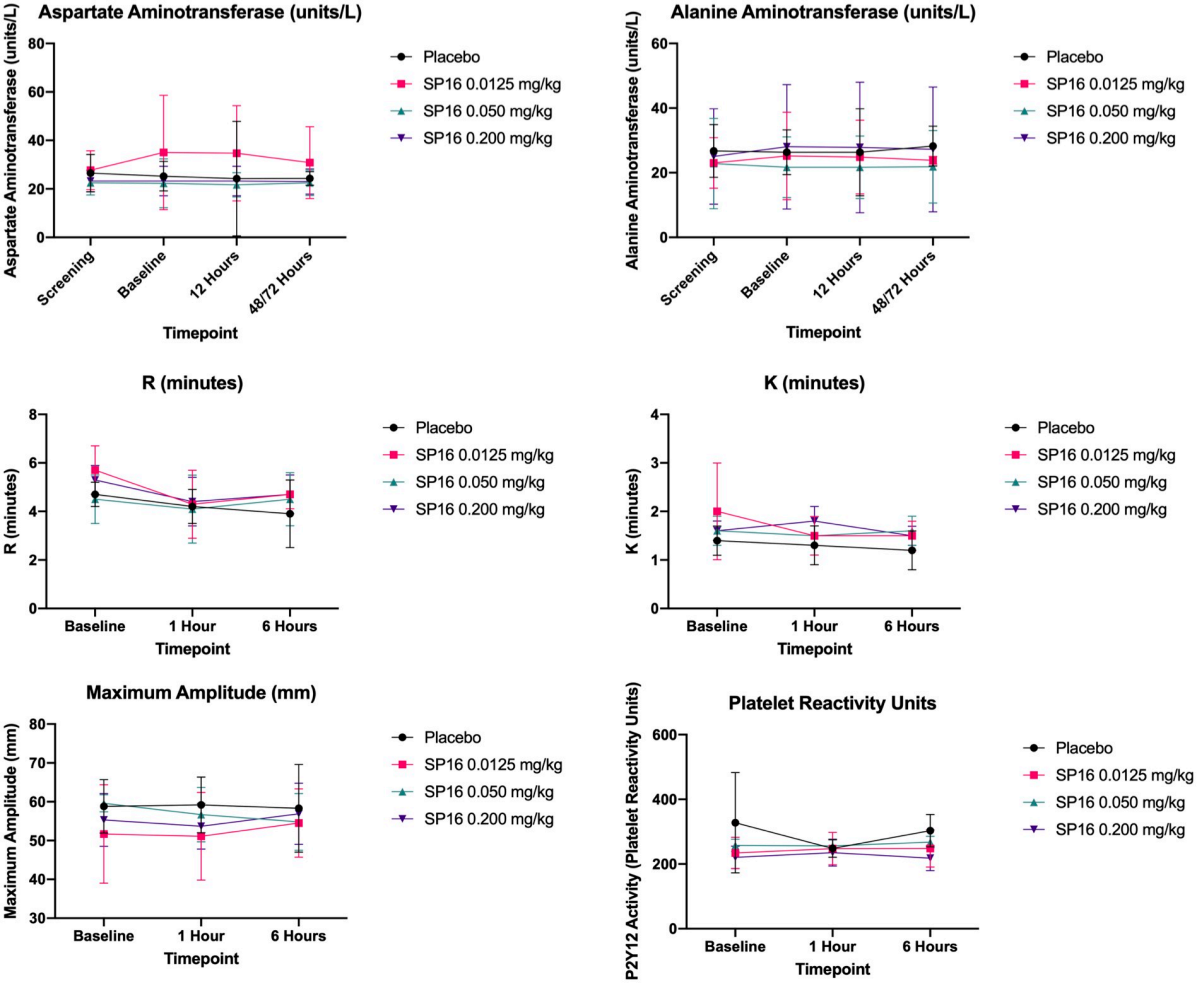
Effects of SP16 on key biochemical safety parameters.

In placebo patients, the median IQR for alanine aminotransferase (ALT) at baseline, 12- and 48/72-hours were 25 (20, 34) units/L, 26 (23, 31) units/L, 29.5 (22, 33) units/L, respectively. In the 0.0125 mg/kg SP16 group ALT levels were 22 (15, 37) units/L, 22 (17, 33) units/L, and 22 (17, 31) units/L, respectively. For the 0.050 mg/kg SP16 cohort the ALT levels were 19 (15, 27) units/L, 19 (16, 25) units/L, and 18 (16, 27) units/L, respectively. And for the 12 mg (0.200 mg/kg) SP16 cohort the ALT levels were 24 (15, 37) units/L, 22 (16, 38) units/L, and 23 (15, 39) units/L, respectively. Analysis of multiple coagulation parameters found no differences between SP16 and placebo. SP16 had no effect on the time to clot formation (R min), the time to 20 mm clot size (K min) or clot strength (maximum amplitude). Similarly, activated partial thromboplastin time and prothrombin time were not different between all three SP16 doses and placebo. Each SP16 dose lacked appreciable effects on P2Y12 receptor-mediated platelet activity (Fig 4).

Analysis of multiple coagulation parameters found no differences between SP16 and placebo. SP16 had no effect on the time to clot formation (R min), the time to 20 mm clot size (K min) or clot strength (maximum amplitude). Similarly, activated partial thromboplastin time and prothrombin time were not different between all three SP16 doses and placebo. Each SP16 dose lacked appreciable effects on P2Y12 receptor-mediated platelet activity.

**Fig 5:**
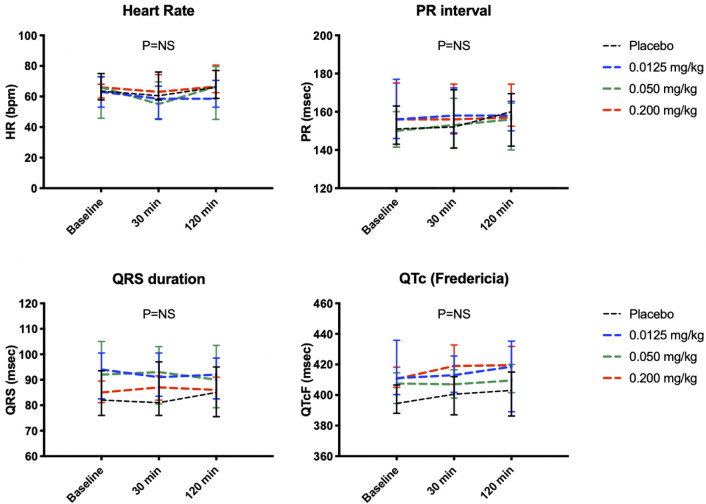
Effects of SP16 on cardiac parameters.

There were no overt signs of myocardial toxicity. Cardiac troponin levels were below detectable limits at all time points in both placebo and SP16 groups. Creatinine kinase-myocardial band levels tended to decrease over time in all groups although none of the changes were statistically significant (see S1 File). There were no abnormalities in heart rate, PR interval, or corrected QT interval on electrocardiogram between groups or within each individual (Fig 5).

Injection site pain was reported by 15 of 16 patients in the SP16 arms and 3 of 6 patients in the placebo arm (P = 0.07). The mean (SD) pain score was 1.5 (2.1) in the placebo arm, 3.5 (2.5) in the SP16 0.0125 mg/kg arm, 4.8 (2.5) in the SP16 0.050 mg/kg arm and 6.0 (1.4) in the 0.200 mg/kg group. The differences between groups were significant by one-way ANOVA (P = 0.011) and the 0.200 mg/kg group experienced significantly more pain than the placebo group (P = 0.0088). No patients reported pain beyond 1/10 after 30 minutes from the injection. There was a dose response noted for injection site pain (R^2^ = 0.293 P = 0.0063). Injection site pain was also correlated with injection volume (R^2^ = 0.174 P = 0.038).

## Discussion

This first-in-human study of SP16, a novel, synthetic LRP1 agonist, demonstrated that a single subcutaneous injection of SP16 up to 0.200 mg/kg (max dose of 12 mg) was well-tolerated and lacked any biochemical effects on coagulation, platelet function and liver enzymes in healthy volunteers. Mild-moderate injection site pain was dose-related and self-resolving in most cases.

SP16 was developed as a synthetic biosuperior to AAT, an endogenous serine protease with anti-inflammatory and pro-survival effects mediated through the LRP1 receptor. SP16 is a 17 amino acid-length peptide that mimics the LRP1 binding site of endogenous serine proteases and is expected to lack off-target effects. A survey of >100 kinases and receptors showed no cross-reactivity of SP16 with other targets. Moreover, anti-SP16 antibodies have not been detected in animals that received up to 6 months of treatment with SP16 and deliberate attempts to produce an anti-SP16 antibody have failed. While this theoretical construct suggests that SP16 offers a meaningful safety advantage over AAT, this first-in-human study tested that hypothesis in healthy volunteers. Our short-term study of a small sample of healthy volunteers suggests that SP16 doses up to 0.200 mg/kg can be administered safely with good tolerability. This study provides reassurances that SP16 may lack many of the adverse effects of AAT, such as hepatic transaminase elevations and infusion reactions. Whether this safety profile extends to adults with acute myocardial infarction, who are older and have multiple comorbidities, remains to be determined.

Most patients in the SP16 arm reported injection site pain at the time of injection and pain severity appeared to be dose-related. Patients who required two separate subcutaneous injections of SP16 to limit the injected volume to less than 2 mL reported significantly greater pain than those who received one injection. Likewise, higher SP16 concentrations also associated with greater pain scores. It is worth noting 92% (22) of patients reported no injection site pain 12 hours after the injection (maximum pain at 12 hours = 1/10) and 100% reported no pain at 24 hours. It will be important to refine the SP16 formulation to remove potentially irritating excipients and increase the injection concentration to limit the number of required injections. An intravenous formulation may avoid injection site reactions altogether. Nevertheless, this adverse effect is not expected to slow clinical development of this promising therapeutic compound.

Although percutaneous coronary intervention in acute myocardial infarction has saved innumerable lives, the resultant ischemia-reperfusion injury remains a source of adverse cardiac remodeling [8]. Data from animal models as well as a pilot study of humans with acute myocardial infarction support the role of LRP1 as a potential treatment for ischemia-reperfusion injury. In the pilot study, 10 patients with ST segment elevation acute myocardial infarction receive a single dose of plasma-derived AAT within 12 hours of hospital admission [9]. In comparison to historical controls, AAT significantly reduced the inflammatory response by day 14. In addition, AAT-treated patients had lower creatine kinase-myocardial band area-under-the-curve compared to historical controls [10], suggesting a beneficial effect on ischemia-reperfusion injury. In mice with experimental acute myocardial infarction, plasma-derived AAT and AAT-Fc fusion protein significantly reduced myocardial inflammation and infarct size, while preserving left ventricular function [11, 12]. Similarly, SP16 reduced infarct size and preserved left ventricular systolic function in a murine model of experimental myocardial infarction at doses as low as 0.1 mg/kg [5]. Importantly, the benefits of SP16 were abrogated by treatment with an LRP1 antibody. This study represents an important step forward in the development of LRP1 agonists, a novel class of anti-inflammatory therapy. Drugs that safely inhibit inflammation are urgently needed across a wide variety of clinical conditions, including the ongoing novel coronavirus 2019 pandemic [13, 14]. An ongoing phase II clinical trial is evaluating SP16 in patients with STEMI to assess the safety and inhibition of the acute inflammatory response [15].

This study has certain limitations, including the small sample size and short duration of follow-up. We also could not test the efficacy or pharmacodynamics of SP16 since all subjects were healthy volunteers with presumably low levels of systemic inflammation. Assay development is ongoing in order to conduct pharmacokinetic analysis of the frozen samples.

## Conclusions

A single subcutaneous injection of SP16, a novel, synthetic LRP1 agonist, was safe and well-tolerated in healthy volunteers. While the lack of overt cardiac (or non-cardiac) toxicities or alterations in coagulation assays is reassuring, future testing is warranted to establish the safety of SP16. Despite these limitations, however, this pilot study provides the foundation for continued development of SP16 and other LRP1 agonists in the treatment of ischemia-reperfusion injury and other acute inflammatory conditions.

## Supporting information

S1 File (DOCX)

S2 File (DOCX)

S1 Checklist CONSORT 2010 checklist of information to include when reporting a randomised trial*. (PDF)
