# Supplementary material for: A phase 1 clinical trial of SP16, a first-in-class anti-inflammatory LRP1 agonist, in healthy volunteers
Source: PLoS One. 2021 May 6;16(5):e0247357. doi: 10.1371/journal.pone.0247357 (PMC8101931; doi:10.1371/journal.pone.0247357)
Supplement: S1 File — (DOCX) [file pone.0247357.s001.docx]

**S1 Table: Safety parameters**

| **White Blood Cells (x10^9^/L): Mean (SD)** | | | |  |  |
| --- | --- | --- | --- | --- | --- |
| **Group** | **Screening** | **Baseline** | **12 hours** | **48-72 hours** | **P-value** |
| Placebo | 7.5 (1.3) | 6.6 (1.1) | 7.8 (1.3) | 7.1 (1.9) | 0.645 |
| SP16 0.0125 mg/kg | 6.0 (1.7) | 5.2 (1.3) | 6.6 (1.4) | 5.5 (1.0) |  |
| SP16 0.050 mg/kg | 6.2 (1.3) | 6.2 (1.3) | 6.8 (1.2) | 5.9 (1.2) |  |
| SP16 0.200 mg/kg | 5.5 (1.3) | 6.3 (1.0) | 7.1 (1.2) | 5.9 (1.0) |  |
| **Red Blood Cells (x109/L): Mean (SD)** | | | |  |  |
| **Group** | **Screening** | **Baseline** | **12 hours** | **48-72 hours** | **P-value** |
| Placebo | 4.8 (0.3) | 4.7 (0.5) | 4.7 (0.6) | 4.7 (0.3) | 0.726 |
| SP16 0.0125 mg/kg | 4.9 (0.6) | 4.6 (0.6) | 4.7 (0.7) | 4.7 (0.6) |  |
| SP16 0.050 mg/kg | 4.6 (0.5) | 4.4 (0.5) | 4.4 (0.4) | 4.5 (0.5) |  |
| SP16 0.200 mg/kg | 5.0 (0.4) | 5.0 (0.4) | 5.0 (0.3) | 4.9 (0.3) |  |
| **Hemoglobin (g/dL): Mean (SD)** | | | |  |  |
| **Group** | **Screening** | **Baseline** | **12 hours** | **48-72 hours** | **P-value** |
| Placebo | 13.7 (1.5) | 13.3 (1.7) | 13.3 (1.8) | 13.3 (1.5) | 0.714 |
| SP16 0.0125 mg/kg | 13.7 (1.7) | 13.1 (1.8) | 13.3 (1.9) | 13.2 (1.7) |  |
| SP16 0.050 mg/kg | 14.2 (1.1) | 13.5 (1.2) | 13.8 (1.1) | 13.7 (1.3) |  |
| SP16 0.200 mg/kg | 14.4 (0.9) | 14.3 (0.8) | 14.2 (0.6) | 14.1 (0.8) |  |
| **Hematocrit (%): Mean (SD)** | | | | |  |
| **Group** | **Screening** | **Baseline** | **12 hours** | **48-72 hours** | **P-value** |
| Placebo | 40.2 (3.2) | 38.9 (4.4) | 38.9 (5.1) | 39.3 (3.1) | 0.711 |
| SP16 0.0125 mg/kg | 41.8 (4.5) | 39.5 (5.1) | 40.2 (5.3) | 39.8 (4.4) |  |
| SP16 0.050 mg/kg | 41.9 (2.7) | 39.7 (3.4) | 40.4 (3.1) | 40.5 (3.2) |  |
| SP16 0.200 mg/kg | 42.4 (3.2) | 42.2 (2.2) | 41.9 (2.0) | 41.7 (2.6) |  |
| **Platelets (x10^9^/L): Mean (SD)** | | | | |  |
| **Group** | **Screening** | **Baseline** | **12 hours** | **48-72 hours** | **P-value** |
| Placebo | 262.2 (36.5) | 253.3 (43.0) | 261.2 (43.8) | 259.7 (50.7) | 0.324 |
| SP16 0.0125 mg/kg | 228.2 (45.2) | 218.8 (43.8) | 233.0 (51.8) | 239.2 (48.0) |  |
| SP16 0.050 mg/kg | 248.5 (41.9) | 215.7 (27.3) | 231.2 (24.1) | 227.8 (32.4) |  |
| SP16 0.200 mg/kg | 247.5 (41.2) | 248.7 (40.9) | 253.6 (45.7) | 248.3 (40.5) |  |
| **Mean Corpuscular Volume (fL): Mean (SD)** | | | | |  |
| **Group** | **Screening** | **Baseline** | **12 hours** | **48-72 hours** | **P-value** |
| Placebo | 83.7 (7.0) | 83.8 (7.4) | 83.8 (6.4) | 84.2 (7.3) | 0.279 |
| SP16 0.0125 mg/kg | 86.3 (6.3) | 85.8 (6.4) | 85.7 (6.7) | 85.8 (6.4) |  |
| SP16 0.050 mg/kg | 92.1 (5.1) | 91.2 (4.1) | 91.1 (4.1) | 91.2 (4.9) |  |
| SP16 0.200 mg/kg | 84.6 (2.8) | 85.0 (2.9) | 84.2 (3.1) | 85.0 (3.4) |  |
| **Mean Corpuscular Hemoglobin (pg): Mean (SD)** | | | | |  |
| **Group** | **Screening** | **Baseline** | **12 hours** | **48-72 hours** | **P-value** |
| Placebo | 28.5 (2.9) | 28.6 (2.9) | 28.6 (2.4) | 28.6 (3.0) | 0.912 |
| SP16 0.0125 mg/kg | 28.2 (2.6) | 28.4 (2.4) | 28.4 (2.5) | 28.4 (2.7) |  |
| SP16 0.050 mg/kg | 31.0 (1.0) | 30.9 (0.9) | 31.0 (0.8) | 30.9 (0.8) |  |
| SP16 0.200 mg/kg | 28.8 (1.7) | 28.7 (1.9) | 28.6 (1.9) | 28.7 (1.8) |  |
| **Mean Corpuscular Hemoglobin Concentration (pg/L): Mean (SD)** | | | | |  |
| **Group** | **Screening** | **Baseline** | **12 hours** | **48-72 hours** | **P-value** |
| Placebo | 34.1 (2.0) | 34.2 (1.4) | 34.2 (0.7) | 33.9 (1.9) | 0.928 |
| SP16 0.0125 mg/kg | 32.7 (1.1) | 33.0 (0.7) | 33.1 (0.7) | 33.1 (1.1) |  |
| SP16 0.050 mg/kg | 33.8 (1.1) | 33.9 (0.7) | 34.1 (0.8) | 33.9 (1.1) |  |
| SP16 0.200 mg/kg | 34.0 (1.4) | 33.8 (1.3) | 34.0 (1.3) | 33.8 (1.0) |  |
| **Red Cell Distribution Width (%): Mean (SD)** | | | | |  |
| **Group** | **Screening** | **Baseline** | **12 hours** | **48-72 hours** | **P-value** |
| Placebo | 13.0 (1.1) | 13.1 (1.2) | 13.1 (1.5) | 13.2 (1.2) | 0.277 |
| SP16 0.0125 mg/kg | 13.6 (1.3) | 13.7 (1.5) | 13.6 (1.3) | 13.6 (1.4) |  |
| SP16 0.050 mg/kg | 12.4 (0.9) | 12.3 (0.8) | 12.3 (0.7) | 12.2 (0.8) |  |
| SP16 0.200 mg/kg | 12.4 (0.7) | 12.3 (0.7) | 12.4 (0.8) | 12.3 (0.7) |  |
| **Mean Platelet Volume (fL): Mean (SD)** | | | | |  |
| **Group** | **Screening** | **Baseline** | **12 hours** | **48-72 hours** | **P-value** |
| Placebo | 10.6 (1.3) | 10.3 (1.3) | 10.5 (0.9) | 10.4 (1.3) | 0.272 |
| SP16 0.0125 mg/kg | 10.8 (0.9) | 10.6 (0.9) | 10.8 (1.0) | 10.5 (0.8) |  |
| SP16 0.050 mg/kg | 10.9 (1.2) | 11.1 (1.1) | 10.9 (0.9) | 11.1 (1.1) |  |
| SP16 0.200 mg/kg | 10.6 (1.1) | 10.4 (1.0) | 10.7 (0.8) | 10.4 (1.0) |  |
| **Sodium (mEq/L): Mean (SD)** | | | | |  |
| **Group** | **Screening** | **Baseline** | **12 hours** | **48-72 hours** | **P-value** |
| Placebo | 138.5 (1.2) | 137.7 (1.9) | 137.2 (1.6) | 137.2 (1.5) | 0.238 |
| SP16 0.0125 mg/kg | 137.5 (3.3) | 138.5 (1.6) | 138.2 (1.9) | 138.3 (1.8) |  |
| SP16 0.050 mg/kg | 138.3 (2.0) | 138.3 (2.2) | 139.8 (1.6) | 138.3 (1.9) |  |
| SP16 0.200 mg/kg | 138.3 (1.8) | 137.5 (1.0) | 138.0 (1.1) | 138.3 (1.2) |  |
| **Potassium (mEq/L): Mean (SD)** | | | | |  |
| **Group** | **Screening** | **Baseline** | **12 hours** | **48-72 hours** | **P-value** |
| Placebo | 4.0 (0.2) | 4.1 (0.4) | 4.1 (0.2) | 4.1 (0.2) | 0.350 |
| SP16 0.0125 mg/kg | 4.3 (0.2) | 3.9 (0.2) | 4.1 (0.3) | 4.4 (0.4) |  |
| SP16 0.050 mg/kg | 4.2 (0.4) | 4.0 (0.2) | 3.9 (0.2) | 4.3 (0.2) |  |
| SP16 0.200 mg/kg | 4.4 (0.3) | 4.0 (0.4) | 4.0 (0.2) | 4.2 (0.3) |  |
| **Chloride (mEq/L): Mean (SD)** | | | | |  |
| **Group** | **Screening** | **Baseline** | **12 hours** | **48-72 hours** | **P-value** |
| Placebo | 105.3 (1.5) | 105.5 (2.4) | 105.0 (1.7) | 104.5 (2.2) | 0.459 |
| SP16 0.0125 mg/kg | 103.8 (2.1) | 105.2 (1.7) | 104.7 (2.1) | 105.5 (1.0) |  |
| SP16 0.050 mg/kg | 104.7 (2.6) | 105.8 (2.6) | 105.7 (1.8) | 105.7 (1.0) |  |
| SP16 0.200 mg/kg | 106.0 (1.7) | 103.8 (1.9) | 104.8 (2.0) | 105.7 (2.3) |  |
| **Carbon Dioxide (x10^9^/L): Mean (SD)** | | | | |  |
| **Group** | **Screening** | **Baseline** | **12 hours** | **48-72 hours** | **P-value** |
| Placebo | 28.0 (3.3) | 28.0 (3.2) | 27.2 (2.6) | 28.7 (2.6) | 0.498 |
| SP16 0.0125 mg/kg | 29.0 (3.4) | 28.8 (2.6) | 29.5 (3.2) | 28.5 (2.1) |  |
| SP16 0.050 mg/kg | 29.3 (3.3) | 28.2 (2.3) | 28.8 (2.6) | 27.7 (1.2) |  |
| SP16 0.200 mg/kg | 28.8 (2.6) | 29.2 (2.0) | 27.8 (1.5) | 28.0 (3.2) |  |
| **Blood Urea Nitrogen (mg/dL): Mean (SD)** | | | | |  |
| **Group** | **Screening** | **Baseline** | **12 hours** | **48-72 hours** | **P-value** |
| Placebo | 12.3 (3.6) | 12.8 (4.6) | 13.7 (3.7) | 11.5 (1.4) | 0.078 |
| SP16 0.0125 mg/kg | 15.3 (3.7) | 17.3 (3.7) | 15.2 (4.3) | 14.5 (3.8) |  |
| SP16 0.050 mg/kg | 14.7 (7.2) | 15.2 (5.2) | 14.5 (4.2) | 17.5 (7.6) |  |
| SP16 0.200 mg/kg | 12.5 (2.7) | 14.2 (2.3) | 13.0 (2.5) | 14.5 (4.5) |  |
| **Serum Creatinine (mg/dL): Mean (SD)** | | | | |  |
| **Group** | **Screening** | **Baseline** | **12 hours** | **48-72 hours** | **P-value** |
| Placebo | 0.8 (0.2) | 0.8 (0.2) | 0.8 (0.2) | 0.8 (0.2) | 0.123 |
| SP16 0.0125 mg/kg | 0.9 (0.2) | 0.8 (0.2) | 0.8 (0.2) | 0.8 (0.1) |  |
| SP16 0.050 mg/kg | 0.7 (0.1) | 0.7 (0.1) | 0.8 (0.1) | 0.8 (0.1) |  |
| SP16 0.200 mg/kg | 0.8 (0.1) | 0.9 (0.0) | 0.8 (0.1) | 0.8 (0.1) |  |
| **Glucose (mg/dL): Mean (SD)** | | | | |  |
| **Group** | **Screening** | **Baseline** | **12 hours** | **48-72 hours** | **P-value** |
| Placebo | 89.5 (8.7) | 90.3 (10.5) | 91.0 (3.5) | 91.0 (14.5) | 0.249 |
| SP16 0.0125 mg/kg | 87.5 (5.0) | 91.8 (3.5) | 89.7 (12.9) | 89.2 (4.6) |  |
| SP16 0.050 mg/kg | 86.2 (10.9) | 85.0 (11.4) | 107.2 (22.9) | 90.2 (13.2) |  |
| SP16 0.200 mg/kg | 88.7 (8.7) | 86.2 (8.2) | 95.0 (17.7) | 94.2 (15.5) |  |
| **Calcium (mEq/L): Mean (SD)** | | | | |  |
| **Group** | **Screening** | **Baseline** | **12 hours** | **48-72 hours** | **P-value** |
| Placebo | 9.7 (0.4) | 9.6 (0.2) | 9.4 (0.3) | 9.7 (0.2) | 0.069 |
| SP16 0.0125 mg/kg | 9.9 (0.1) | 9.5 (0.3) | 9.8 (0.3) | 9.8 (0.2) |  |
| SP16 0.050 mg/kg | 9.9 (0.2) | 9.7 (0.2) | 9.6 (0.3) | 9.8 (0.4) |  |
| SP16 0.200 mg/kg | 9.6 (0.4) | 9.7 (0.3) | 9.7 (0.3) | 9.7 (0.2) |  |
| **Alkaline Phosphatase (units/L): Mean (SD)** | | | | |  |
| **Group** | **Screening** | **Baseline** | **12 hours** | **48-72 hours** | **P-value** |
| Placebo | 82.0 (18.6) | 79.7 (16.0) | 79.8 (7.6) | 81.3 (21.1) | 0.202 |
| SP16 0.0125 mg/kg | 56.5 (9.0) | 54.0 (7.6) | 54.2 (8.6) | 53.8 (8.0) |  |
| SP16 0.050 mg/kg | 62.7 (22.1) | 62.3 (23.5) | 62.5 (21.5) | 62.8 (22.8) |  |
| SP16 0.200 mg/kg | 72.2 (20.7) | 72.0 (19.1) | 73.8 (19.7) | 63.3 (30.2) |  |
| **Aspartate Aminotransferase (units/L): Mean (SD)** | | | | |  |
| **Group** | **Screening** | **Baseline** | **12 hours** | **48-72 hours** | **P-value** |
| Placebo | 26.5 (7.7) | 25.2 (6.1) | 24.2 (23.6) | 24.3 (2.8) | 0.613 |
| SP16 0.0125 mg/kg | 27.7 (8.0) | 35.0 (23.6) | 34.7 (19.7) | 30.8 (14.8) |  |
| SP16 0.050 mg/kg | 22.5 (5.0) | 21.3 (3.6) | 21.7 (5.0) | 22.5 (5.2) |  |
| SP16 0.200 mg/kg | 23.2 (4.4) | 22.3 (10.1) | 23.2 (6.1) | 23.0 (5.2) |  |
| **Alanine Aminotransferase (units/L): Mean (SD)** | | | | |  |
| **Group** | **Screening** | **Baseline** | **12 hours** | **48-72 hours** | **P-value** |
| Placebo | 26.7 (8.2) | 26.3 (6.9) | 26.3 (13.5) | 28.2 (6.2) | 0.773 |
| SP16 0.0125 mg/kg | 23.0 (7.8) | 25.2 (13.5) | 24.8 (11.4) | 23.8 (10.2) |  |
| SP16 0.050 mg/kg | 22.8 (14.0) | 21.7 (9.4) | 21.7 (9.7) | 21.8 (11.2) |  |
| SP16 0.200 mg/kg | 25.0 (14.8) | 28.0 (19.3) | 27.8 (20.2) | 27.2 (19.3) |  |
| **Total Protein (g/dL): Mean (SD)** | | | | |  |
| **Group** | **Screening** | **Baseline** | **12 hours** | **48-72 hours** | **P-value** |
| Placebo | 7.5 (0.7) | 7.1 (0.7) | 7.0 (0.3) | 7.3 (0.5) | 0.622 |
| SP16 0.0125 mg/kg | 7.7 (0.4) | 7.1 (0.3) | 7.3 (0.3) | 7.4 (0.4) |  |
| SP16 0.050 mg/kg | 7.5 (0.3) | 7.1 (0.2) | 7.2 (0.3) | 7.3 (0.4) |  |
| SP16 0.200 mg/kg | 7.3 (0.6) | 7.2 (0.7) | 7.1 (0.5) | 7.2 (0.4) |  |
| **Albumin (g/dL): Mean (SD)** | | | | |  |
| **Group** | **Screening** | **Baseline** | **12 hours** | **48-72 hours** | **P-value** |
| Placebo | 4.6 (0.4) | 4.3 (0.4) | 4.2 (0.2) | 4.4 (0.4) | 0.773 |
| SP16 0.0125 mg/kg | 4.7 (0.1) | 4.5 (0.2) | 4.5 (0.3) | 4.7 (0.2) |  |
| SP16 0.050 mg/kg | 4.7 (0.3) | 4.5 (0.2) | 4.4 (0.2) | 4.5 (0.3) |  |
| SP16 0.200 mg/kg | 4.5 (0.3) | 4.5 (0.3) | 4.4 (0.2) | 4.4 (0.2) |  |
| **Total Bilirubin (g/dL): Mean (SD)** | | | | |  |
| **Group** | **Screening** | **Baseline** | **12 hours** | **48-72 hours** | **P-value** |
| Placebo | 0.8 (0.2) | 0.5 (0.1) | 0.4 (0.1) | 0.6 (0.1) | 0.768 |
| SP16 0.0125 mg/kg | 0.6 (0.3) | 0.5 (0.1) | 0.4 (0.1) | 0.6 (0.2) |  |
| SP16 0.050 mg/kg | 0.8 (0.2) | 0.7 (0.2) | 0.5 (0.2) | 0.7 (0.3) |  |
| SP16 0.200 mg/kg | 0.5 (0.2) | 0.6 (0.2) | 0.4 (0.1) | 0.6 (0.2) |  |
| **Estimated Glomerular Filtration Rate (mL/min/1.73m^2^): Mean (SD)** | | | | |  |
| **Group** | **Screening** | **Baseline** | **12 hours** | **48-72 hours** | **P-value** |
| Placebo | 107.7 (9.8) | 108.5 (13.3) | 106.3 (7.7) | 110.5 (8.9) | 0.507 |
| SP16 0.0125 mg/kg | 109.2 (11.9) | 114.3 (7.7) | 115.0 (7.4) | 113.0 (8.3) |  |
| SP16 0.050 mg/kg | 112.0 (10.4) | 109.6 (11.3) | 109.0 (11.4) | 107.8 (13.4) |  |
| SP16 0.200 mg/kg | 115.2 (5.2) | 111.8 (7.5) | 114.3 (8.7) | 114.3 (7.7) |  |
| **Anion Gap (mEq/L): Mean (SD)** | | | | |  |
| **Group** | **Screening** | **Baseline** | **12 hours** | **48-72 hours** | **P-value** |
| Placebo | 5.2 (2.5) | 4.2 (1.5) | 5.0 (0.8) | 4.0 (2.4) | 0.951 |
| SP16 0.0125 mg/kg | 4.7 (3.3) | 4.5 (0.8) | 4.0 (2.0) | 4.3 (1.4) |  |
| SP16 0.050 mg/kg | 4.3 (1.6) | 4.3 (1.8) | 5.3 (2.6) | 5.0 (1.9) |  |
| SP16 0.200 mg/kg | 3.5 (1.6) | 4.5 (1.0) | 5.3 (2.5) | 4.7 (1.6) |  |
| **Globulin (g/dL): Mean (SD)** | | | | |  |
| **Group** | **Screening** | **Baseline** | **12 hours** | **48-72 hours** | **P-value** |
| Placebo | 2.9 (0.4) | 3.2 (1.0) | 2.8 (0.2) | 2.8 (0.4) | 0.288 |
| SP16 0.0125 mg/kg | 2.9 (0.4) | 2.6 (0.2) | 2.8 (0.3) | 2.8 (0.3) |  |
| SP16 0.050 mg/kg | 2.8 (0.2) | 2.6 (0.2) | 2.8 (0.2) | 2.7 (0.3) |  |
| SP16 0.200 mg/kg | 2.8 (0.3) | 2.7 (0.4) | 2.7 (0.3) | 2.8 (0.2) |  |
| **R (min): Mean (SD)** | | | | |  |
| **Group** | **Baseline** | **1 hour** | **6 hours** | **P-value** |  |
| Placebo | 4.7 (0.5) | 4.2 (0.7) | 3.9 (1.4) | 0.496 |  |
| SP16 0.0125 mg/kg | 5.7 (1.0) | 4.3 (1.4) | 4.7 (0.6) |  |  |
| SP16 0.050 mg/kg | 4.5 (1.0) | 4.1 (1.4) | 4.5 (1.1) |  |  |
| SP16 0.200 mg/kg | 5.3 (0.6) | 4.4 (1.0) | 4.7 (0.8) |  |  |
| **K (min): Mean (SD)** | | | | |  |
| **Group** | **Baseline** | **1 hour** | **6 hours** | **P-value** |  |
| Placebo | 1.4 (0.3) | 1.3 (0.4) | 1.2 (0.4) | 0.201 |  |
| SP16 0.0125 mg/kg | 2.0 (1.0) | 1.5 (0.4) | 1.5 (0.3) |  |  |
| SP16 0.050 mg/kg | 1.6 (0.3) | 1.5 (0.4) | 1.6 (0.3) |  |  |
| SP16 0.200 mg/kg | 1.6 (0.2) | 1.8 (0.3) | 1.5 (0.2) |  |  |
| **Angle (degrees): Mean (SD)** | | | | |  |
| **Group** | **Baseline** | **1 hour** | **6 hours** | **P-value** |  |
| Placebo | 69.5 (3.4) | 71.0 (5.3) | 71.8 (5.8) | 0.369 |  |
| SP16 0.0125 mg/kg | 63.1 (9.6) | 69.0 (5.8) | 69.1 (3.9) |  |  |
| SP16 0.050 mg/kg | 67.9 (3.1) | 68.1 (4.7) | 68.5 (3.7) |  |  |
| SP16 0.200 mg/kg | 66.4 (3.1) | 67.0 (3.8) | 69.0 (2.6) |  |  |
| **Maximum Amplitude (mm): Mean (SD)** | | | | |  |
| **Group** | **Baseline** | **1 hour** | **6 hours** | **P-value** |  |
| Placebo | 58.8 (6.9) | 59.2 (7.2) | 58.3 (11.3) | 0.794 |  |
| SP16 0.0125 mg/kg | 51.7 (12.7) | 51.1 (11.3) | 54.5 (8.8) |  |  |
| SP16 0.050 mg/kg | 59.6 (2.2) | 56.9 (7.0) | 54.8 (7.3) |  |  |
| SP16 0.200 mg/kg | 55.3 (6.8) | 53.7 (5.9) | 56.9 (7.9) |  |  |
| **Activated Partial Thromboplastin Time (sec): Mean (SD)** | | | | |  |
| **Group** | **Baseline** | **1 hour** | **6 hours** | **P-value** |  |
| Placebo | 31.8 (2.8) | 31.8 (2.3) | 32.0 (2.3) | 0.589 |  |
| SP16 0.0125 mg/kg | 31.8 (3.3) | 31.7 (2.3) | 32.3 (2.7) |  |  |
| SP16 0.050 mg/kg | 29.5 (2.9) | 29.0 (2.8) | 29.3 (3.1) |  |  |
| SP16 0.200 mg/kg | 31.7 (3.8) | 31.5 (4.1) | 29.0 (8.5) |  |  |
| **P2Y12 Activity (Platelet Reactivity Units): Mean (SD)** | | | | |  |
| **Group** | **Baseline** | **1 hour** | **6 hours** | **P-value** |  |
| Placebo | 327.8 (155) | 248.2 (26.8) | 303.0 (50.3) | 0.585 |  |
| SP16 0.0125 mg/kg | 234.2 (48.6) | 247.8 (50.3) | 248.2 (57.6) |  |  |
| SP16 0.050 mg/kg | 257.7 (19.2) | 256.0 (18.9) | 267.8 (18.2) |  |  |
| SP16 0.200 mg/kg | 220.5 (34.9) | 235.3 (41.7) | 218.0 (38.5) |  |  |
| **Prothrombin Time (sec): Mean (SD)** | | | | |  |
| **Group** | **Baseline** | **1 hour** | **6 hours** | **P-value** |  |
| Placebo | 13.6 (0.6) | 13.7 (0.4) | 13.7 (0.4) | 0.269 |  |
| SP16 0.0125 mg/kg | 13.4 (0.5) | 13.7 (0.4) | 13.7 (0.3) |  |  |
| SP16 0.050 mg/kg | 13.6 (0.5) | 13.6 (0.5) | 13.6 (0.6) |  |  |
| SP16 0.200 mg/kg | 13.6 (0.5) | 14.0 (0.4) | 13.6 (0.2) |  |  |
| **International Normalized Ratio (ratio units): Mean (SD)** | | | | |  |
| **Group** | **Baseline** | **1 hour** | **6 hours** | **P-value** |  |
| Placebo | 1.1 (0.1) | 1.1 (0.1) | 1.0 (0.0) | 0.119 |  |
| SP16 0.0125 mg/kg | 1.1 (0.1) | 1.1 (0.0) | 1.1 (0.0) |  |  |
| SP16 0.050 mg/kg | 1.1 (0.1) | 1.1 (0.1) | 1.1 (0.1) |  |  |
| SP16 0.200 mg/kg | 1.0 (0.1) | 1.1 (0.1) | 1.0 (0.0) |  |  |
| **High Sensitivity Troponin I (ng/mL): Mean (SD)** | | | | |  |
| **Group** | **Baseline** | **6 hours** | **12 hours** | **P-value** |  |
| Placebo | 0.03 (0.0) | 0.03 (0.0) | 0.03 (0.0) | N/A |  |
| SP16 0.0125 mg/kg | 0.03 (0.0) | 0.03 (0.0) | 0.03 (0.0) |  |  |
| SP16 0.050 mg/kg | 0.03 (0.0) | 0.03 (0.0) | 0.03 (0.0) |  |  |
| SP16 0.200 mg/kg | 0.03 (0.0) | 0.03 (0.0) | 0.03 (0.0) |  |  |
| *0.03 ng/mL = Lower Detection Limit  **Creatine Kinase-Myocardial Band (ng/mL): Mean (SD)** | | | | |  |
| **Group** | **Baseline** | **12 hours** | **48-72 hours** | **P-value** |  |
| Placebo | 1.0 (0.4) | 0.8 (0.3) | 0.7 (0.8) | 0.834 |  |
| SP16 0.0125 mg/kg | 1.7 (0.9) | 1.5 (0.8) | 1.3 (0.7) |  |  |
| SP16 0.050 mg/kg | 1.0 (0.9) | 0.8 (0.5) | 0.8 (0.6) |  |  |
| SP16 0.200 mg/kg | 1.2 (0.6) | 1.0 (0.6) | 0.9 (0.6) |  |  |
| **Heart rate (per minute): Mean (SD)** | | | | |  |
| **Group** | **Baseline** | **30 min** | **120 min** | **P-value** |  |
| Placebo | 65.0 (8.6) | 65.0 (9.6) | 66.8 (12.1) | 0.604 |  |
| SP16 0.0125 mg/kg | 61.8 (12.2) | 56.7 (12.1) | 59.5 (11.1) |  |  |
| SP16 0.050 mg/kg | 61.5 (13.9) | 57.7 (13.5) | 63.8 (18.0) |  |  |
| SP16 0.200 mg/kg | 64.3 (5.4) | 65.0 (10.6) | 69.8 (11.5) |  |  |
| **PR Interval (msec): Mean (SD)** | | | | |  |
| **Group** | **Baseline** | **30 min** | **120 min** | **P-value** |  |
| Placebo | 152.3 (16.2) | 155.0 (20.8) | 157.0 (11.9) | 0.819 |  |
| SP16 0.0125 mg/kg | 160.0 (15.5) | 159.3 (11.9) | 157.7 (9.1) |  |  |
| SP16 0.050 mg/kg | 149.7 (12.8) | 151.7 (20.9) | 152.3 (14.4) |  |  |
| SP16 0.200 mg/kg | 157.7 (18.2) | 159.7 (15.0) | 161.7 (12.7) |  |  |
| **QRS Interval (msec): Mean (SD)** | | | | |  |
| **Group** | **Baseline** | **30 min** | **120 min** | **P-value** |  |
| Placebo | 85.0 (10.9) | 85.7 (12.0) | 86.7 (11.6) | 0.491 |  |
| SP16 0.0125 mg/kg | 90.7 (13.1) | 90.3 (11.6) | 90.0 (10.4) |  |  |
| SP16 0.050 mg/kg | 93.0 (14.5) | 92.0 (14.6) | 91.0 (15.5) |  |  |
| SP16 0.200 mg/kg | 85.3 (5.5) | 86.7 (6.7) | 86.3 (5.6) |  |  |
| **QTc Fredericia (msec): Mean (SD)** | | | | |  |
| **Group** | **Baseline** | **30 min** | **120 min** | **P-value** |  |
| Placebo | 395.5 (12.1) | 398.5 (16.3) | 400.8 (23.6) | 0.698 |  |
| SP16 0.0125 mg/kg | 417.0 (19.9) | 415.8 (23.6) | 414.3 (23.6) |  |  |
| SP16 0.050 mg/kg | 405.5 (13.7) | 408.0 (10.3) | 408.8 (13.2) |  |  |
| SP16 0.200 mg/kg | 411.7 (8.5) | 419.8 (14.8) | 421.7 (9.3) |  |  |

**S2 Table: Safety parameter testing schedule**

| **Safety Parameter** | **Screen** | **Time (hours)** | | | | | | |
| --- | --- | --- | --- | --- | --- | --- | --- | --- |
|  |  | **0** | **0.5** | **1** | **2** | **6** | **12** | **48-72** |
| Complete Blood Count (CBC)   - WBC, RBC, HGB, HCT, PLT, MCV, MCH, MCHC, RDW, MPV | X | X |  |  |  |  | X | X |
| Comprehensive Metabolic Panel   - Na, K, Cl, CO2, BUN, SCr, Glu, Ca, eGFR, AnionGap | X | X |  |  |  |  | X | X |
| Liver Function Test   - AST, ALT, AlkP, TProt, Alb, TBili, Glob | X | X |  |  |  |  | X | X |
| Thromboelastography   - K, R, Angle, MaxAmp |  | X |  | X |  | X |  |  |
| Coagulation   - aPTT, PT, INR, P2Y12 |  | X |  | X |  | X |  |  |
| Biomarkers   - hsTnI, CK-MB |  | X |  |  |  | X | X |  |
| Electrocardiogram   - HR, PR, QRS, QTcF |  | X | X |  | X |  |  |  |
| WBC = White blood cell; RBC = Red blood cell; HGB = Hemoglobin; HCT = Hematocrit; PLT = Platelet; MCV = Mean Corpuscular Volume; MCH = Mean Corpuscular Hemoglobin; MCHC = Mean Corpuscular Hemoglobin Concentration; Red blood cell Distribution Width; MPV = Mean Platelet Volume; Na = Sodium; K = Potassium; Cl = Chloride; CO2 = Carbon Dioxide; BUN = Blood Urea Nitrogen; SCr = Serum Creatinine; Glu = Glucose; Ca = Calcium; eGFR = estimated GFR; AST = Aspartate Aminotransferase; ALT = Alanine Aminotransferase; AlkP = Alkaline Phosphatase; TProt = Total Protein; Alb = Albumin; TBili = Total Bilirubin; Glob = Globulin; MaxAmp = Maximum Amplitude; aPTT = activated Partial Thromboplastin Time; PT = Prothombin Time; INR = International Normalized Ratio; P2Y12 = P2Y12 Platelet Reactivity; hsTnI = High Sensitivity Troponin I; CK-MB = Creatine Kinase-Myoglobal Band; HR = Heart Rate; PR = PR interval; QRS = QRS duration; QTcF = QT correction (Fredericia) | | | | | | | | |
