# Supplementary material for: A phase 1 clinical trial of SP16, a first-in-class anti-inflammatory LRP1 agonist, in healthy volunteers
Source: PLoS One. 2021 May 6;16(5):e0247357. doi: 10.1371/journal.pone.0247357 (PMC8101931; doi:10.1371/journal.pone.0247357)
Supplement: S2 File — (DOCX) [file pone.0247357.s002.docx]

**Safety, Tolerability and Pharmacokinetics of a Single Subcutaneous Administration of SP16–a SERPIN- like, Small Peptide Agonist of the Low Density Lipoprotein-like Receptor 1–in Healthy Individuals**

**Protocol Identifying Number: 01-SP16**

**Principal Investigators: Antonio Abbate, MD, PhD**

**Benjamin W. Van Tassell, PharmD**

**IND/IDE Sponsor: Serpin Pharma, LLC**

**Version Number: v.3 10 July 2018**

**Table of Contents**

# TABLE OF CONTENTS

[LIST OF ABBREVIATIONS 4](#_bookmark0)

[STATEMENT OF COMPLIANCE 6](#_bookmark1)

[PROTOCOL SUMMARY 7](#_bookmark2)

1. [KEY ROLES 9](#_bookmark3)
2. [INTRODUCTION: BACKGROUND INFORMATION AND SCIENTIFIC RATIONALE 10](#_bookmark4)
   1. [BACKGROUND INFORMATION 10](#_bookmark5)
   2. [RATIONALE 11](#_bookmark6)
   3. [POTENTIAL RISKS AND BENEFITS 12](#_bookmark7)
      1. [KNOWN POTENTIAL RISKS 12](#_bookmark8)
      2. [KNOWN POTENTIAL BENEFITS 12](#_bookmark9)
3. [OBJECTIVES AND PURPOSE 12](#_bookmark10)
4. [STUDY DESIGN AND ENDPOINTS 12](#_bookmark11)
   1. [DESCRIPTION OF THE STUDY DESIGN 12](#_bookmark12)
   2. [STUDY ENDPOINTS 13](#_bookmark13)
      1. [PHARMACOKINETIC ENDPOINTS 13](#_bookmark14)
5. [STUDY ENROLLMENT AND WITHDRAWAL 13](#_bookmark15)
   1. [PARTICPANT INCLUSION CRITERIA 13](#_bookmark16)
   2. [PARTICIPANT EXCLUSION CRITERA 13](#_bookmark17)
   3. [STRATEGIES FOR RECRUITMENT AND RETENTION 14](#_bookmark18)
   4. [PARTICIPANT WITHDRAWAL OR TERMINATION 14](#_bookmark19)
      1. [REASONS FOR WITHDRAWAL OR TERMINATION 14](#_bookmark20)
      2. [HANDLING OF PARTICIPANT WITHDRAWALS OR TERMINATION 14](#_bookmark21)
   5. [PREMATURE TERMINATION OR SUSPENSION OF STUDY 14](#_bookmark22)
6. [STUDY AGENT 15](#_bookmark23)
   1. [STUDY AGENT(S) AND CONTROL DESCRIPTION 15](#_bookmark24)
      1. [ACQUISITION 15](#_bookmark25)
      2. [FORMULATION, APPEARANCE, PACKAGING, AND LABELING 15](#_bookmark26)
      3. [PRODUCT STORAGE AND STABILITY 15](#_bookmark27)
      4. [PREPARATION 15](#_bookmark28)
      5. [DOSING AND ADMINISTRATION 16](#_bookmark29)
      6. [ROUTE OF ADMINISTRATION 16](#_bookmark30)
      7. [STARTING DOSE AND DOSE ESCALATION SCHEDULE 16](#_bookmark31)
      8. [DURATION OF THERAPY 16](#_bookmark32)
      9. [TRACKING OF DOSE 16](#_bookmark33)
   2. [STUDY AGENT ACCOUNTABILITY PROCEDURES 16](#_bookmark34)
7. [STUDY PROCEDURES AND SCHEDULE 17](#_bookmark35)
   1. [STUDY PROCEDURES/EVALUATIONS 17](#_bookmark36)
      1. [STUDY SPECIFIC PROCEDURES 17](#_bookmark37)
      2. [STANDARD OF CARE STUDY PROCEDURES 17](#_bookmark38)
   2. [LABORATORY PROCEDURES/EVALUATIONS 17](#_bookmark39)
      1. [CLINICAL LABORATORY EVALUATIONS 17](#_bookmark40)
      2. [OTHER ASSAYS OR PROCEDURES 17](#_bookmark41)
      3. [SPECIMEN PREPARATION, HANDLING, AND STORAGE 18](#_bookmark42)
      4. [SPECIMEN SHIPMENT 18](#_bookmark43)
   3. [STUDY SCHEDULE 18](#_bookmark44)
      1. [SCREENING (VISIT 0) 18](#_bookmark45)
      2. [BASELINE (VISIT 1) 18](#_bookmark46)
      3. [FOLLOW-UP 19](#_bookmark47)
      4. [FINAL STUDY VISIT 19](#_bookmark48)

2

- - 1. [EARLY TERMINATION VISIT 19](#_bookmark49)
    2. [UNSCHEDULED VISIT 19](#_bookmark50)
    3. [SCHEDULE OF EVENTS TABLE 20](#_bookmark51)
  1. [CONCOMITANT MEDICATIONS, TREATMENTS, AND PROCEDURES 20](#_bookmark52)
  2. [JUSTIFICATION FOR SENSITIVE PROCEDURES 20](#_bookmark53)
     1. [PRECAUTIONARY MEDICATIONS, TREATMENTS, AND PROCEDURES 20](#_bookmark54)
  3. [PROHIBITED MEDICATIONS, TREATMENTS, AND PROCEDURES 20](#_bookmark55)
  4. [PROPHYLACTIC MEDICATIONS, TREATMENTS, AND PROCEDURES 20](#_bookmark56)
  5. [RESCUE MEDICATIONS, TREATMENTS, AND PROCEDURES 20](#_bookmark57)
  6. [PARTICIPANT ACCESS TO STUDY AGENT AT STUDY CLOSURE 20](#_bookmark58)

1. [ASSESSMENT OF SAFETY 21](#_bookmark59)
   1. [SPECIFICATION OF SAFETY PARAMETERS 21](#_bookmark60)
      1. [DEFINITION OF ADVERSE EVENTS (AE) 21](#_bookmark61)
      2. [DEFINITION OF SERIOUS ADVERSE EVENTS (SAE) 21](#_bookmark62)
      3. [DEFINITION OF UNANTICIPATED PROBLEMS (UP) 21](#_bookmark63)
   2. [CLASSIFICATION OF AN ADVERSE EVENT 22](#_bookmark64)
      1. [SEVERITY OF EVENT 22](#_bookmark65)
      2. [RELATIONSHIP TO STUDY AGENT 22](#_bookmark66)
      3. [EXPECTEDNESS 23](#_bookmark67)

[8.2 TIME PERIOD AND FREQUENCY FOR EVENT ASSESSMENT AND FOLLOW-UP 23](#_bookmark68)

- - 1. [ADVERSE EVENT REPORTING 23](#_bookmark69)
    2. [SERIOUS ADVERSE EVENT REPORTING 23](#_bookmark70)
    3. [UNANTICIPATED PROBLEM REPORTING 24](#_bookmark71)
    4. [EVENTS OF SPECIAL INTEREST 24](#_bookmark72)
    5. [REPORTING OF PREGNANCY 24](#_bookmark73)
  1. [STUDY HALTING RULES 24](#_bookmark74)
  2. [SAFETY OVERSIGHT 25](#_bookmark75)

1. [CLINICAL MONITORING 25](#_bookmark76)
2. [STATISTICAL CONSIDERATIONS 25](#_bookmark77)
   1. [ANALYSIS DATASETS 25](#_bookmark78)
   2. [DESCRIPTION OF STATISTICAL METHODS 25](#_bookmark79)
      1. [GENERAL APPROACH 25](#_bookmark80)
   3. [MEASURES TO MINIMIZE BIAS 25](#_bookmark81)
      1. [ENROLLMENT / RANDOMIZATION / MASKING PROCEDURES 25](#_bookmark82)
      2. [BREAKING THE STUDY BLIND/PARTICIPANT CODE 26](#_bookmark83)
3. [SOURCE DOCUMENTS AND ACCESS TO SOURCE DATA/DOCUMENTS 26](#_bookmark84)
4. [QUALITY ASSURANCE AND QUALITY CONTROL 26](#_bookmark85)
5. [ETHICS/PROTECTION OF HUMAN SUBJECTS 26](#_bookmark86)
   1. [ETHICAL STANDARD 26](#_bookmark87)
   2. [INSTITUTIONAL REVIEW BOARD 26](#_bookmark88)
   3. [INFORMATED CONSENT PROCESS 26](#_bookmark89)
      1. [CONSENT/ASSENT AND OTHER INFORMATIONAL DOCUMENTS PROVIDED TO PARTICIPANTS 26](#_bookmark90)
      2. [CONSENT PROCEDURES AND DOCUMENTATION 26](#_bookmark91)
   4. [PARTICIPANT AND DATA CONFIDENTIALITY 27](#_bookmark92)
      1. [RESEARCH USE OF STORED HUMAN SAMPLES, SPECIMENS OR DATA 27](#_bookmark93)
   5. [FUTURE USE OF STORED SPECIMENS 27](#_bookmark94)
6. [DATA HANDLING AND RECORD KEEPING 27](#_bookmark95)
   1. [DATA COLLECTION AND MANAGEMENT RESPONSIBILITIES 27](#_bookmark96)
   2. [STUDY RECORDS RETENTION 27](#_bookmark97)
   3. [PROTOCOL DEVIATIONS 28](#_bookmark98)
   4. [PUBLICATION AND DATA SHARING POLICY 28](#_bookmark99)
7. [STUDY ADMINISTRATION 28](#_bookmark100)
   1. [STUDY LEADERSHIP 28](#_bookmark101)
8. [CONFLICT OF INTEREST POLICY 28](#_bookmark102)
9. [APPENDIX 29](#_bookmark103)
10. [LITERATURE REFERENCES 30](#_bookmark104)

LIST OF ABBREVIATIONS

| Abbreviation | Expanded Term |
| --- | --- |
| λ | Terminal rate constant |
| AAT | α1-Anti-Trypsin |
| AE | Adverse event |
| ALT | Alanine aminotransferase |
| AMI | Acute myocardial infarction |
| AST | Aspartate aminotransferase |
| AUC∞ | Total area-under-the-curve |
| CFR | Code of Federal Regulations |
| CHD | Coronary heart disease |
| CLtot | Total clearance |
| cmax | Maximum concentration |
| CRSU | Clinical Research Service Unit |
| DSMB | Data Safety Monitoring Board |
| ECG | Electrocardiogram |
| FDA | Food and Drug Administration |
| GCP | Good Clinical Practice |
| IDS | Investigational Drug Services |
| IRB | Institutional Review Board |
| LRP1 | Low Density Lipoprotein Receptor-like Protein 1 |
| MSRD | Maximum Recommended Starting Dose |
| MRTsys | Mean resident time |
| PI | Principle Investigator |
| SAE | Serious adverse event |
| SERPINS | Serine protease inhibitors |
| SP16 | Serpin peptide 16 |
| STEMI | ST-segment elevation |
| t1/2 | Half-life |
| tmax | Time to Cmax |
| TNF | Tumor necrosis factor |
| UP | Unanticipated problems |

| US | United States |
| --- | --- |
| VCU | Virginia Commonwealth University |
| Vdcc | Volume of distribution of the central compartment |
| Vdss | Volume of distribution at steady-state |
| WBC | White blood cell |

STATEMENT OF COMPLIANCE

The trial will be carried out in accordance with Good Clinical Practice (GCP) as required by United States (US) Code of Federal Regulations (CFR) applicable to clinical studies (45 CFR Part 46, 21 CFR Part 50, 21 CFR Part 56, 21 CFR Part 312, and/or 21 CFR Part 812) and International Conference on Harmonisation Guidance for Industry, Good Clinical Practice: Consolidated Guidance.

All key personnel (all individuals responsible for the design and conduct of this trial) have completed Human Subjects Protection Training.

I agree to ensure that all staff members involved in the conduct of this study are informed about their obligations in meeting the above commitments.

Principal Investigator: Antonio Abbate, MD, PhD

Print/Type Name

Principal Investigator: Benjamin Van Tassell, PharmD

Print/Type Name

Signed:

Date/Signature

Date/Signature

PROTOCOL SUMMARY

| Title: | Safety, Tolerability and Pharmacokinetics of a Single Subcutaneous Administration of SP16–a SERPIN-like, Small Peptide Agonist of the Low Density Lipoprotein Receptor-like Protein 1 (LRP1)- in Healthy Individuals |
| --- | --- |
| Design: | This study is a randomized, double-blind, placebo-controlled Phase 1 Clinical Trial of 24 healthy individuals who will be randomized in a 3:1 allocation ratio to receive either a single administration of either SP16 or matching placebo into one of three (0.0125, 0.050 or 0.200 mg/kg) dose groups. In each dosing cohort, three patients (2 active and 1 placebo) will be randomized to receive a single study drug infusion on separate days, at least 24 hours apart. The remaining subjects in each dosing cohort (1 placebo and 4 active) will receive study drug after at least 24 hours from the 3rd subject. |
| Objectives: | The primary objective of this study is to determine the safety and tolerability of a single subcutaneous administration of SP16 in healthy individuals. The secondary objective is to determine the pharmacokinetics in these subjects. |
| Study Endpoints: | Pharmacokinetic analysis and safety assessments will be performed. |
| Safety Monitoring: | Subjects will be monitored directly for 12-hours after administration of investigational medication or placebo and then have repeated follow-up at 24 hours, 48-72 hours and 7 days. An unblinded safety assessment will be performed after the first 3 subjects of each group have been monitored for 24 hours. Advancement to the final 5 patients of each dosing group will be allowed only upon review of the first 3 subjects and exclusion of treatment related adverse events with a given dose [sentinel dosing]. Subsequent dosing groups will be initiated after the safety assessment of the previous group is complete (see **Figure 1** below). |
| Pharmacokinetic Considerations: | Pharmacokinetic parameters include total clearance, maximum concentration, time to maximum concentration, mean resident time, volume of distribution of the central compartment, volume of distribution at pseudo-steady-state, volume of distribution at steady-state, total area-under-the-curve, half-life, and terminal rate constant. |
| Population: | Individuals (N = 24) will be included if they are at least 18 years of age and healthy. |
| Phase: | 1 |
| Number of Sites Enrolling Participants | 1 (Virginia Commonwealth University) |
| Description of | SP16 is a synthetic oligopeptide based upon the structure of human alpha-1 |

| Study Agent: | antitrypsin. SP16 exhibits high affinity for the LRP1. |
| --- | --- |
| Study Duration: | 6 months |
| Participant Duration: | 1 week |

***Figure 1: Schematic of the Study Design***

**SP16 0.0125 mg/kg or matching placebo**

>24 hr

>24 hr

>24 hr

**Subject 1 Subject 2 Subject 3**

**Subjects 4, 5, 6, 7, 8**

>24 hr

>24 hr

>24 hr

**Subject 1 Subject 2 Subject 3**

**Subjects 4, 5, 6, 7, 8**

>24 hr

>24 hr

>24 hr

**Subject 1 Subject 2 Subject 3**

**Subjects 4, 5, 6, 7, 8**

**Unblinded Safety Assessment**

**Unblinded Safety Assessment**

**Unblinded Safety Assessment**

**Unblinded Safety Assessment**

**Unblinded Safety Assessment**

**SP16 0.05 mg/kg once or matching placebo**

**SP16 0.2 mg/kg once or matching placebo**

1. KEY ROLES Principal Investigators:

Antonio Abbate, MD, PhD

James C. Roberts, Esq., Professor in Cardiology Department of Internal Medicine

Virginia Commonwealth University 1200 E Broad Street

Richmond, VA 23298

Benjamin Van Tassell, PharmD

Vice Chair for Clinical Research and Associate Professor Department of Pharmacotherapy & Outcomes Science Virginia Commonwealth University

410 North 12th Street, Rm 636

Richmond, VA 23298

1. INTRODUCTION: BACKGROUND INFORMATION AND SCIENTIFIC RATIONALE
   1. BACKGROUND INFORMATION

Serpin Peptide 16 (SP16) is a synthetic anti-inflammatory peptide developed to reproduce

the -anti-inflammatory and α activities of the Serine Protease Inhibitors (SERPINs or Serpins), such as 1-Anti-Trypsin (AAT). Serpins are a family of proteins characterized by the ability to inhibit plasma serine proteases such as elastase, thrombin, plasmin.**1** When Serpins bind to serine proteases with resulting inactivation of enzymatic activity, causing a conformational change by which a short peptide containing a unique motif (5-11 amino-acids) is exposed.**2,3** This motif binds to LRP1, a membrane receptor responsible for clearance of plasma proteins and for inducing an anti-inflammatory and cyto protective signal.[3](#_bookmark105)

Serpin Pharma has developed a synthetic anti-inflammatory peptide (“SP16”) that contains the properties of the Serpin core motif responsible for the anti-inflammatory and cyto-protective signaling, without inhibiting the plasma serine proteases (Issued Patent #8,975,224). SP16 is 17 amino acids in length, derived by excision of a 36-39 amino acid long peptide fragment to yield an active short peptide containing the unique motif. SP16 represents the shortest oligopeptide maintaining activity and a single amino acid substitution enhancing anti-inflammatory signaling and plasma stability. In vitro, SP16 did not exhibit meaningful affinity for receptors other than LRP-1.

From a safety perspective, SP16 appears to bind exclusively to LRP-1. A survey of >100 kinases and receptors showed no cross-reactivity of SP16 with other targets. SP16 appears not to be immunogenic. Antibodies to SP16 have not been detected in animal models after up to 6 months of treatment. Furthermore, deliberate attempts to produce anti-SP16 monoclonal antibodies failed to provoke an immune response.

In comparison with endogenous Serpins, SP16 is a bio-superior- small peptide which retains the anti-inflammatory and cyto-protective properties of Serpins. SP16 only contains the LRP-1 binding motif, thus providing specific anti-inflammatory actions without serine protease inhibition.

SP16 has demonstrated anti-inflammatory properties in several pre-clinical models. In ex vivo peripheral blood mononuclear cells derived from a collagen antibody induced arthritis mouse model, SP16 significantly reduced LPS-stimulated tumor necrosis factor (TNF)-α and Interleukin (IL)-6 production compared to vehicle and no treatment. SP16 has also been tested in db-/db diabetic mice, in which it significantly reduces serum amyloid A, an acute phase reactant, and monocyte chemotactic protein-1. Moreover, SP16 reduced IL-6 concentrations through inhibition of Myd88-mediated NF-κB activation, a key inflammatory signaling pathway. In vitro experiments have also demonstrated that SP16 inhibits NFκB activation.**4**

SP16 has been studied extensively in non-clinical toxicology studies. A single subcutaneous administration of SP16 at doses up to 60 mg/kg in Sprague-Dawley rats with a 14-day recovery period was well tolerated. There were no test article-related findings on clinical observation, body weights, body weight changes, food consumption and ophthalmology. There were no test article- related findings on hematology, coagulation, urinalysis, absolute and relative organ weights or macroscopic examinations. Test article- related findings were limited to increased incidence of slight edema injection site in males given 60 mg/kg, minimal decreases in albumin in males given 60 mg/kg, minimal increases in total cholesterol and triglycerides in females given ≥ 20 mg/kg, and microscopic changes at the injection site -a mild but increased severity of inflammation occurred in animals given 60 mg/kg, eosinophilic material and mild necrosis in subcutis of animals

given ≥5 mg/kg). The mild necrosis in the subcutis noted in males administered 60 mg/kg and in females administered ≥5 mg/kg was considered adverse.

- 1. RATIONALE

Despite advances in prevention and treatment, coronary heart disease (CHD) is still the leading cause of mortality and morbidity worldwide. CHD kills more than 385,000 people annually and each year about 935,000 Americans have an acute myocardial infarction (AMI).**5** Despite current strategies for early reperfusion, many patients die early during the course, and those who survive are at risk for dying later from adverse cardiac remodeling, heart failure and sudden death. The major determinants for outcome in patients with AMI are the size of infarct, the degree of the inflammatory response and the remaining left ventricular systolic function.**6** Patients presenting with ST-segment elevation (STEMI) are at particularly high risk for in-hospital death, adverse cardiac remodeling, heart failure, and long-term mortality.**4** Improving the treatment of acute myocardial infarction to prevent heart failure and death remains an urgent unmet medical need.

SP16 has been tested as treatment for acute myocardial infarction in the mouse. AMI was induced by temporary ligation of the proximal left coronary artery for 30 minutes. Mice which had received a single administration of SP16 immediately at reperfusion or within 30 minutes of reperfusion had smaller infarct sizes and better left ventricular systolic function at 24 hours and 7 days.

The main hypothesis of this study is that a single subcutaneous administration of SP16 is safe and well tolerated in healthy individuals.

SP16 will be administered subcutaneously as this route has greater ease of administration than intravenous injection. A single dose administration has been selected based upon pre-clinical data and expected clinical use of SP16.

The initial SP16 dose (level 1) was chosen according to FDA Guidance for the calculation of the Maximum Recommended Starting Dose (MSRD)**7** and previous experience with an analogous compound with promiscuous binding to several serine proteases (plasma-derived α1-antitrypsin) in a Phase II trial of 10 patients with ST-segment elevation myocardial infarction.**8** In that study, plasma-derived α1-antitrypsin was administered at a dose of 60 mg/kg, which is equimolar to an SP16 dose of 2 mg/kg. This 60 mg/kg dose of plasma-derived α1-antitrypsin was well- tolerated.[**7**](#_bookmark106)[**,8**](#_bookmark107)Thus, the initial starting SP16 dose of 0.0125 mg/kg is less than an equivalent dose of plasma-derived α1-antitrypsin which was -well tolerated in a 10 patient Phase 1 clinical trial.

The MSRD was also calculated based on non-clinical safety studies. The initial SP16 starting dose and was performed as follows. In rats, doses of up to 60 mg/kg were administered without toxicity. The dose of 60 mg/kg was then divided by the Food and Drug Administration (FDA) Human Equivalent Dose Conversion Factor of 6.2, yielding a Human Equivalent Dose of 9.677 mg/kg of SP16. Using the FDA-recommended safety factor of 10, an initial starting SP16 dose of 0.97 mg/kg would be considered to have acceptable safety. Thus, the initial starting SP16 dose in this study (0.0125 mg/kg) exceeds the FDA standards with a safety factor of 0.97/0.0125 = 77.4. Since there has been no observable toxicity below 60 mg/kg in pre-clinical animal models, it is possible that the MSRD calculated for this protocol underestimates the true MSRD. Nevertheless, the initial dose was chosen to maximize patient safety.

- 1. POTENTIAL RISKS AND BENEFITS
     1. KNOWN POTENTIAL RISKS

Allergic reaction to any drug, including SP16, is possible, and severe allergic reactions can be life threatening. In clinical trials, the most common adverse reactions to plasma derived AAT (to which SP16 is related) occurring in >1% subjects were headache (7%), musculoskeletal discomfort (myalgias) (7%) and sore throat (pharyngitis) (2%). Rash, hot flushing and pruritus (itching) may also occur (<1%). All these reactions were considered mild or moderate and not severe and were self-limiting with the end of the infusion[.**8**](#_bookmark107) An elevation in the aminotransferase levels (ALT or AST) may occur (10%) deriving from subtle alterations in the muscles and/or liver. Such elevations were minor (less than 5-times baseline), transient (resolve within 3 months) and did not result in symptoms or any long-term consequences. Similar to any drug or intervention, the possibility of an unanticipated adverse reaction cannot be excluded.

There is a possibility that an assessment completed during the screening phase will reveal an unrecognized disease or condition (i.e. pregnancy). The results of the tests will be discussed with the subject, and if requested, forwarded to a chosen physician.

Loss of confidentiality is a potential risk. The likelihood of this occurring, however, is very low. The database will not directly link subjects’ identity with their clinical data. In addition, except when required by law, subjects will not be identified by name, social security number, address, telephone number or any other personal identifier. Study related tests may be reported but subjects will not be identified.

There is minimal risk associated with the physical procedures of the study. The electrocardiogram is no greater than minimal risk. The blood draws may result in minor bleeding or discomfort (rare) and infection (extremely rare) at puncture site. An indwelling peripheral venous catheter will be placed by appropriately trained personnel on study day 1 to facilitate frequent blood draws while minimizing patient discomfort and risk.

There are no expected direct medical benefits to the study participants. All participants will be compensated for their participation.

Defining the safety profile of SP16 in healthy subjects is necessary to continue clinical development of SP16 as a novel therapeutic for patients with STEMI. This necessity justifies the potential risks to human subjects.

- - 1. KNOWN POTENTIAL BENEFITS There are no known benefits of SP16.

1. OBJECTIVES AND PURPOSE

The primary objective of this study is to assess the safety and tolerability of a single subcutaneous dose of SP16.

The secondary objective of this study is to define the pharmacokinetic parameters of a single subcutaneous dose of SP16 in healthy subjects.

1. STUDY DESIGN AND ENDPOINTS
   1. DESCRIPTION OF THE STUDY DESIGN

This study is a single-center, Phase 1, randomized, double-blind, placebo-controlled, dose escalation study of SP16 in healthy individuals. Subjects will be randomized to receive either SP16 or matching placebo in a 3:1 allocation ratio. Within the first 8 subjects, 6 will be randomized to

SP16 and will receive a dose of 0.0125 mg/kg once and 2 will receive a matching volume of placebo. Within the next 8 subjects, 6 will be randomized to SP16 and will receive a dose of 0.050 mg/kg once and 2 will receive a matching volume of placebo. In the last group of 8 subjects, 6 will be randomized to SP16 and will receive a dose of 0.200 mg/kg once and 2 will receive a matching volume of placebo.

- 1. STUDY ENDPOINTS

The primary objective of the study is the safety of SP16 including assessment of potential adverse events, laboratory abnormalities, or electrocardiogram abnormalities.

- - 1. PHARMACOKINETIC ENDPOINTS

Pharmacokinetic data will be analyzed using non-compartmental analysis. This approach will yield estimates of pharmacokinetic parameters such as total clearance (CLtot), cmax (maximum concentration), tmax (time to Cmax), mean resident time (MRTsys), volume of distribution of the central compartment (Vdcc), volume of distribution at pseudo-steady-state (Vdpss), volume of distribution at steady-state (Vdss), total area-under-the-curve (AUC∞), half-life (t1/2), and terminal rate constant (λ) without having to specify a particular compartment model.

1. STUDY ENROLLMENT AND WITHDRAWAL
   1. PARTICPANT INCLUSION CRITERIA

In order to be eligible to participate in this study, an individual must meet all of the following criteria:

- Written informed consent
- Age 18 to 59 years
- Stated willingness to comply with all study procedures and availability for the duration of the study
- Ability to take oral medication and be willing to adhere to the medication regimen
- For females of reproductive potential: Use of highly effective contraception
- For males of reproductive potential: Use of condoms
  1. PARTICIPANT EXCLUSION CRITERA

An individual who meets any of the following criteria will be excluded from participation in this study:

- Acute or chronic illness affecting organ function or requiring medications (including, but not limited to, cardiovascular, hepatic, renal hematologic, neurologic, dermatologic, psychiatric, or rheumatologic disease);
- Febrile illness within the previous 14 days;
- Known allergic reactions to components of the study agent;
- Treatment with another investigational drug or other intervention within 30 days;
- Current tobacco use or tobacco use within 60 days;
- Household contacts who are immunocompromised;
- Chronic infection(s) (of any kind);
- Malignancy (of any kind);
- Substance abuse disorder(s);
- Pregnancy or breastfeeding;
- Any other conditions that would place the subject at increased risk of adverse events or interfere with the conduct or interpretation of the study, in the opinion of the investigators.
  1. STRATEGIES FOR RECRUITMENT AND RETENTION

Recruitment will occur at Virginia Commonwealth University (VCU) at an expected rate of 1 subject per week. Recruitment will occur via word-of-mouth and displays of recruitment material. Recruitment flyers will be reviewed and approved by the IRB and they will include information about the study, subject compensation and the investigators’ contact information.

All study candidates will undergo screening to rule out any pre-existing health conditions. Pre- screening procedures will include a history, physical exam, vital signs, electrocardiogram, pregnancy test (if appropriate), laboratory tests including comprehensive metabolic panel and complete blood cell count. Subjects who meet any of the exclusion criteria (above) will not be eligible for participation.

Participants will be compensated with a total of $700 for their time and effort related to participation in this study. They will receive a Visa Gift card with value of $500 after Visit 1 and

$200 after Visit 3.

- 1. PARTICIPANT WITHDRAWAL OR TERMINATION

Participants may withdraw voluntarily from the study or the Principal Investigator (PI) may terminate a participant from the study.

- - 1. REASONS FOR WITHDRAWAL OR TERMINATION

Participants are free to withdraw from participation in the study at any time. An investigator may terminate participation in the study if:

- - - - Any clinical adverse event (AE), laboratory abnormality, or other medical condition or situation occurs such that continued participation in the study would not be in the best interest of the participant
      - The participant meets an exclusion criterion (either newly developed or not previously recognized) that precludes further study participation.

Halting criteria for study conduct are described separately in Sections 5.5 and 8.5.

- - 1. HANDLING OF PARTICIPANT WITHDRAWALS OR TERMINATION

Effort will be made to complete follow-up for all participants who receive blinded investigational medication to ascertain safety endpoints. Participants who withdraw from the study prior to receipt of blinded investigational medication will be considered screen failures and this enrollment spot will be replaced by another subject. Participants who receive blinded investigational medication but refuse all forms of follow-up will be encouraged to seek alternative medical follow-up.

- 1. PREMATURE TERMINATION OR SUSPENSION OF STUDY

This study may be temporarily suspended or prematurely terminated if there is sufficient reasonable cause. The Sponsor and Principal Investigators will jointly evaluate study progress and risks to subjects after the first 3 subjects have completed a given dose level and prior to escalation to the next dose level. Written notification, documenting the reason for study suspension or termination, will be provided by the suspending or terminating party to regulatory authorities. If the study is prematurely terminated or suspended, the PI will promptly inform the Institutional Review Board (IRB) and will provide the reason(s) for the termination or suspension.

Circumstances that may warrant termination or suspension include, but are not limited to:

- Determination of unexpected, significant, or unacceptable risk to participants
- Data that are not sufficiently complete and/or evaluable

Study may resume once concerns about safety, protocol compliance, and data quality are addressed and satisfy the sponsor, IRB and/or FDA.

Specific halting rules are described in Section 8.5.

1. STUDY AGENT
   1. STUDY AGENT(S) AND CONTROL DESCRIPTION
      1. ACQUISITION

SP16 will be supplied by the Sponsor Serpin Pharma LLC to the Investigational Drug Services (IDS) Pharmacy of Virginia Commonwealth University.

- - 1. FORMULATION, APPEARANCE, PACKAGING, AND LABELING

Investigational SP16 will be prepared by Serpin Pharma LLC. Vials of investigational medication or placebo will be identical in appearance and SP16 will be indistinguishable from placebo. The pharmacy at the clinical site (VCU) will transfer the drug (SP16) from the vial into a tube in preparation for the injection. A placebo solution (identical to the formulation used for SP16, without SP16) will be prepared by the pharmacy and transferred into a tube in preparation for the injection.

- - 1. PRODUCT STORAGE AND STABILITY

The drug is stored in 4.5 mL, brown vials in -20oC. The drug is formulated in water, pH 5.8-6.2, at 3 mg/mL. Final volume in each vial is 2.5 mL. The drug was manufactured for phase I clinical trial on Aug 25, 2017 and will undergo stability testing after 3, 6, 9 and 12 months post manufacturing. The API stability after 12 months since manufacturing was found to be essentially unchanged.


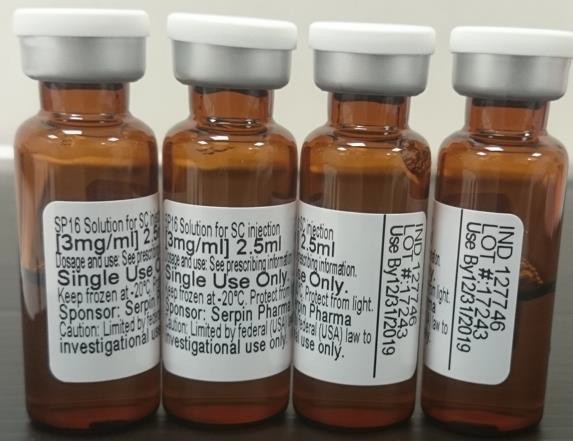


- - 1. PREPARATION

SP16 API was re-suspended in double distilled water and pH was adjusted to pH 5.9 ± 0.02 by adding 0.1M hydrochloric acid and/or 0.1M sodium hydroxide solution slowly. For complete details of formulation and sterile-fill-finish, please see MTA 172-2 and BMR-991.

- - 1. DOSING AND ADMINISTRATION

The drug can be thawed up to 6 hours from the time of injection and can be stored afterwards at 4oC until injection. Unused drug should be discarded and not be used for additional patients.

Each subject will be randomized to receive either a single, weight-based dose of SP16 or matching placebo. All doses will be administered as a subcutaneous injection in the CRSU on Visit 1.

The first 6 subjects who are randomized to SP16 will receive a single dose of 0.0125 mg/kg. The next 6 subjects randomized to SP16 will receive 0.050 mg/kg. The last 6 subjects randomized to SP16 will receive 0.200 mg/kg.

- - 1. ROUTE OF ADMINISTRATION

SP16 or matching placebo will be administered as a subcutaneous injection.

- - 1. STARTING DOSE AND DOSE ESCALATION SCHEDULE

The dose levels of investigational medication (SP16 or matching placebo) will be

- - - - Dose Level 1: SP16 0.0125 mg/kg (up to a maximum of 12 mg) or matching placebo
      - Dose Level 2: SP16 0.050 mg/kg (up to a maximum of 12 mg) or matching placebo
      - Dose Level 3: SP16 0.200 mg/kg (up to a maximum of 12 mg) or matching placebo

SP16 is formulated as a 2-mL syringe containing 6 mg of SP16 (3 mg/mL), such that some patients will require 2 separate subcutaneous injections to deliver the total dose.

A total of 8 subjects will be randomized within each Dose Level in an allocation ratio of 3:1 such that 6 subjects receive active SP16 and 2 subjects receive matching placebo. The first 3 subjects in each Dose Level will be enrolled at least 24 hours apart to allow for sentinel monitoring of at least one subject. A sentinel period of 3 subjects is required due to the randomization allocation ratio of 3:1. After the first three subjects have completed 24-hours of follow-up, the Principal Investigators and Sponsor will review unblinded safety data and adverse events, if any, and jointly determine whether to continue enrollment of the remaining 5 subjects within that Dose Level. Enrollment of the final 5 subjects within a Dose Level can occur without time restrictions.

After all subjects within a dose level have completed follow-up, the Principal Investigators and Sponsor will review unblinded safety data and adverse events, if any, and jointly determine whether to advance to the next Dose Level.

- - 1. DURATION OF THERAPY

All subjects in group 1 and 2 will receive a single dose administration while subjects in group 3 would receive the full dose in 2 site injections. Repeat doses will not be administered.

- - 1. TRACKING OF DOSE

Investigational drug storage will be recorded and maintained by the VCU IDS Pharmacy. Dose administration will be recorded electronically by the bedside nurse.

- 1. STUDY AGENT ACCOUNTABILITY PROCEDURES

The VCU Investigational Drug Services Pharmacy will store and dispense all doses of investigational SP16 or matching placebo. Tracking of all incoming doses, dispensed doses, administered dose and wasted doses will be handled by the IDS Pharmacy according to local and national regulations. A single shipment of 24 investigational doses will be delivered by Serpin Pharma LLC to the IDS Pharmacy prior to the start of enrollment. Any unused syringes will be returned to Serpin Pharma LLC at the end of the study period.

1. STUDY PROCEDURES AND SCHEDULE
   1. STUDY PROCEDURES/EVALUATIONS
      1. STUDY SPECIFIC PROCEDURES

The following research procedures will be performed.

- - - - Medical history through patient interview, and if available, medical record review by a licensed study physician.
      - Medication history as reported by the patient, and if available, through pharmacy refill records by a licensed study physician.
      - A complete review of systems and physical examination will be performed by a licensed study physician.
      - 12-lead electrocardiogram (ECG) will be administered by a registered nurse or ECG technician and interpreted by a licensed study physician at baseline, 30 minutes, and 120 minutes after drug administration. ECGs will be used to evaluate changes in heart rate (HR), P-R interval, QRS duration, QT interval, Fridericia-corrected QT interval (QTc), ST segment deviations and T-wave abnormalities. **9**
      - Blood sample collection for comprehensive metabolic panel, complete blood cell count, coagulation tests, cardiac injury markers and pharmacokinetics will be performed by a registered nurse. Blood samples will be drawn from an indwelling venous catheter.
    1. STANDARD OF CARE STUDY PROCEDURES There are no standard of care procedures.
  1. LABORATORY PROCEDURES/EVALUATIONS A Schedule of Procedures is included in Section 7.3.7.
     1. CLINICAL LABORATORY EVALUATIONS The following laboratory evaluations will be performed.
- Hematology: hemoglobin, hematocrit, white blood cells (WBC) with differential count, platelet count.
  - Screening and after enrollment
- Biochemistry: sodium, potassium, carbon dioxide, chloride, blood urea nitrogen, creatinine, total bilirubin, total protein, albumin, alanine aminotransferase (ALT), aspartate aminotransferase (AST), alkaline phosphatase.
  - Screening and after enrollment
- Cardiac injury markers: high-sensitivity cardiac troponin T, creatine kinase-MB
  - After enrollment only
- Coagulation assays: prothrombin time, activated partial thromboplastin time, and thromboelastography [selected times]
- Platelet function testing (P2Y12 receptor function)
- Pharmacokinetics: t1/2, Cmax, Tmax, AUC, clearance
  - After enrollment only
- Pregnancy test
  - Screening only
    1. OTHER ASSAYS OR PROCEDURES None.
    2. SPECIMEN PREPARATION, HANDLING, AND STORAGE

Blood samples will be drawn from a venous catheter and stored at -80 C prior to analysis. Each sample will be labeled with the subject’s unique study identifier.

- - 1. SPECIMEN SHIPMENT

The drug will be shipped on dry ice using GMP procedures (Global Courier) from the manufacturing site in the UK to the pharmacy at VCU. The entire drug supply for phase I would arrive in one shipment. The boxes and vials are labeled (see label).

**SP16 Solution for SQ injection [3mg/ml] 2.5ml**

Dosage and use: See prescribing information.

Single Use Only.

Keep frozen at -20°C. Protect from light. Sponsor: Serpin Pharma

Caution: Limited by federal (USA) law to investigational use only.

IND 127746

LOT #:12743

Use By12/31/2019

- 1. STUDY SCHEDULE
     1. SCREENING (VISIT 0)

# Screening (Visit 0): Day -14 to -1

- - - - Review of all study procedures and answer any participant questions
      - Obtain informed consent of potential participant verified by signature on written informed consent for screening form.
      - Collect demographic information
      - Review medical history to determine eligibility based on inclusion/exclusion criteria.
      - Review medications history to determine eligibility based on inclusion/exclusion criteria.
      - Perform medical examinations needed to determine eligibility based on inclusion/exclusion criteria.
        - Physical examination, including vital signs
        - ECG
      - Collect blood for comprehensive metabolic panel, complete blood cell count and, if appropriate, a pregnancy test.
      - Schedule study visits for participants who are eligible and available for the duration of the study.
    1. BASELINE (VISIT 1) Baseline Visit (Visit 1, Day 0)
       - Re-verify that subject meets inclusion/exclusion criteria.
         - History and physical examination, including vital signs
         - Electrocardiogram
       - Admission to the VCU CRSU
       - Placement of an intravenous cannula (18G-20G) in the arm or hand
       - Vital signs will be monitored at baseline, and then every 15 +/- 5 minutes for the first 1

hour after the study drug infusion and again at 90 +/- 5, 120 +/- 5, 180 +/- 5 minutes, 6 +/-

0.5, and 12 +/- 1

- - - - Safety blood samples will be obtained at baseline and 12 hours after investigational drug administration and will include a comprehensive metabolic panel, a complete blood cell count and markers for cardiac injury.
      - Blood samples for SP16 pharmacokinetic analysis will be obtained at baseline, 15 +/- 5, 30

+/- 5, 45 +/- 5, 60 +/- 5, 90 +/- 5, 120 +/- 5, 180 +/- 5minutes, and at 6 +/- 0.5 and 12 +/- 1 hours after investigational drug administration.

- - - - Blood samples for markers of cardiac injury will be repeated at 6 and 12 hours after investigational drug administration.
      - Blood samples for assessment of hemostasis and platelet function will be obtained at baseline, 60 +/- 5 minutes and 6 +/- 0.5 hours after investigational drug administration.
    1. FOLLOW-UP Visit 2 (24 hours)
       - Vital signs will be monitored at 24 +/- 4 hours
       - Record adverse events as reported by participant
    2. FOLLOW-UP Visit 3 (48-72 hours)
       - Subject interview for adverse events.
       - Record adverse events as reported by participant.
    3. FINAL STUDY VISIT (Visit 4, Day 7 +/- 2)
       - Record AEs as reported by participant or observed by investigator.
       - Record vital signs, results of history and physical examination.
    4. EARLY TERMINATION VISIT

In the event that subject wishes to discontinue participation after receipt of investigational drug but before the final schedule visit (Visit 4), Visit 4 procedures will be performed at the time the subject chooses to withdraw if he/she is willing.

- - 1. UNSCHEDULED VISIT There will be no unscheduled visits.
    2. SCHEDULE OF EVENTS TABLE

| Procedures | | Screening | Baseline (Visit 1) | Follow-up (Visit 2) | Follow-up (Visit 3) | Final Visit (Visit 4) |
| --- | --- | --- | --- | --- | --- | --- |
| Informed consent | | X |  |  |  |  |
| Verify subject meets inclusion/exclusion | | X | X |  |  |  |
| Demographics | | X |  |  |  |  |
| Medical history and physical examination | | X | X | X |  | X |
| Randomization | |  | X |  |  |  |
| CBC w/diff, plts | | X | X |  | X |  |
| Comprehensive metabolic panel a | | X | X |  | X |  |
| Serum Pregnancy test b | | X |  |  |  |  |
| EKG (as indicated) | | X | X |  |  |  |
| Administer SP16 or matching placebo | |  | X |  |  |  |
| Cardiac injury markers | |  | X |  |  |  |
| Coagulation assays | |  | X |  |  |  |
| Pharmacokinetic analysis | |  | X |  |  |  |
| Subject interview for adverse events | |  |  | X | X | X |
| Record adverse events if and as reported by | |  |  | X | X | X |
|  | a: Albumin, alkaline phosphatase, total bilirubin, bicarbonate, BUN, calcium, chloride, creatinine, glucose, LDH, phosphorus, potassium, total protein, SGOT [AST], SGPT [ALT],sodium.  b: Serum pregnancy test (women of childbearing potential). | | | | | |

- 1. CONCOMITANT MEDICATIONS, TREATMENTS, AND PROCEDURES

As all subjects will be healthy and lack indications for concomitant medication use, no concomitant medications will be allowed at the time of enrollment. This stipulation, however, may be waived for subjects who require concomitant medications, treatments or procedures to manage events that occur during the study.

- 1. JUSTIFICATION FOR SENSITIVE PROCEDURES

The use of placebo control is required to determine whether any adverse events that occur during the study are attributable to SP16. Loss of potential benefit is not relevant as all subjects are healthy volunteers and there are no known benefits of SP16.

- - 1. PRECAUTIONARY MEDICATIONS, TREATMENTS, AND PROCEDURES None.
  1. PROHIBITED MEDICATIONS, TREATMENTS, AND PROCEDURES

Treatment with any prescription or non-prescription medications will not be permitted unless discussed with and approved by a licensed study physician, with the exception of problems that emerge during the conduct of the study.

- 1. PROPHYLACTIC MEDICATIONS, TREATMENTS, AND PROCEDURES Prophylactic medications, treatments or procedures will not be administered.
  2. RESCUE MEDICATIONS, TREATMENTS, AND PROCEDURES

Rescue medications, treatments and procedures, such as anti-histamines to treat a rash, may be administered at the discretion of a licensed study physician in order to treat problems that emerge during the conduct of the study. There will be no restrictions with respect to the type or nature of rescue medications, treatments and procedures, which will be chosen at the discretion of a licensed study physician.

- 1. PARTICIPANT ACCESS TO STUDY AGENT AT STUDY CLOSURE

Not applicable.

1. ASSESSMENTS OF SAFETY
   1. SPECIFICATION OF SAFETY PARAMETERS

All AEs that occur during the study will be recorded. At each study visit, subjects will be interviewed to identify any AEs that have occurred. In addition, physical examinations, laboratory tests and other pre-specified assessments will be evaluated to identify AEs.

Study halting rules and AE definitions are described elsewhere (Section 8.1.1, 8.1.2 and 8.5). Pre- specified safety reviews are described in Section 6.1.7.

Risks to subjects will be minimized by restricting study procedures to appropriately trained personnel.

- - 1. DEFINITION OF ADVERSE EVENTS (AE)

Adverse event means any untoward medical occurrence associated with the use of an intervention in humans, whether or not considered intervention-related (21 CFR 312.32 (a)).

Note: Laboratory/ECG/vital signs abnormalities are not an AE in themselves unless clinically relevant or meet the definition of an AE.

- - 1. DEFINITION OF SERIOUS ADVERSE EVENTS (SAE)

Serious adverse event or serious suspected adverse reaction. An AE or suspected adverse reaction is considered "serious" if, in the view of either the investigator or sponsor, it results in any of the following outcomes: death, a life-threatening adverse event, inpatient hospitalization or prolongation of existing hospitalization, a persistent or significant incapacity or substantial disruption of the ability to conduct normal life functions, or a congenital anomaly/birth defect.

Important medical events that may not result in death, be life-threatening, or require hospitalization may be considered serious when, based upon appropriate medical judgment, they may jeopardize the patient or subject and may require medical or surgical intervention to prevent one of the outcomes listed in this definition. Examples of such medical events include allergic bronchospasm requiring intensive treatment in an emergency room or at home, blood dyscrasias or convulsions that do not result in inpatient hospitalization, or the development of drug dependency or drug abuse.

- - 1. DEFINITION OF UNANTICIPATED PROBLEMS (UP)

OHRP considers unanticipated problems involving risks to participants or others to include, in general, any incident, experience, or outcome that meets **all** of the following criteria:

- Unexpected in terms of nature, severity, or frequency given (a) the research procedures that are described in the protocol-related documents, such as the IRB-approved research protocol and informed consent document; and (b) the characteristics of the participant population being studied;
- Related or possibly related to participation in the research (“possibly related” means there is a reasonable possibility that the incident, experience, or outcome may have been caused by the procedures involved in the research); and
- Suggests that the research places participants or others at a greater risk of harm (including physical, psychological, economic, or social harm) than was previously known or recognized.

This study will use the OHRP definition of UP.

- 1. CLASSIFICATION OF AN ADVERSE EVENT
     1. SEVERITY OF EVENT

For AEs not included in the protocol defined grading system, the following guidelines will be used to describe severity.

- **Mild** – Events require minimal or no treatment and do not interfere with the participant’s daily activities.
- **Moderate** – Events result in a low level of inconvenience or concern with the therapeutic measures. Moderate events may cause some interference with functioning.
- **Severe** – Events interrupt a participant’s usual daily activity and may require systemic drug therapy or other treatment. Severe events are usually potentially life- threatening or incapacitating.
  - 1. RELATIONSHIP TO STUDY AGENT

For all collected AEs, the clinician who examines and evaluates the participant will determine the AE’s causality based on temporal relationship and his/her clinical judgment. The degree of certainty about causality will be graded using the categories below.

- **Definitely Related** – There is clear evidence to suggest a causal relationship, and other possible contributing factors can be ruled out. The clinical event, including an abnormal laboratory test result, occurs in a plausible time relationship to drug administration and cannot be explained by concurrent disease or other drugs or chemicals. The response to withdrawal of the drug (de-challenge) should be clinically plausible. The event must be pharmacologically or phenomenologically definitive, with use of a satisfactory re- challenge procedure if necessary.
- **Probably Related** – There is evidence to suggest a causal relationship, and the influence of other factors is unlikely. The clinical event, including an abnormal laboratory test result, occurs within a reasonable time after administration of the drug, is unlikely to be attributed to concurrent disease or other drugs or chemicals, and follows a clinically reasonable response on withdrawal (de-challenge). Re-challenge information is not required to fulfill this definition.
- **Possibly Related** – There is some evidence to suggest a causal relationship (e.g., the event occurred within a reasonable time after administration of the trial medication). However, other factors may have contributed to the event (e.g., the participant’s clinical condition, other concomitant events). Although an AE may rate only as “possibly related” soon after discovery, it can be flagged as requiring more information and later be upgraded to “probably related” or “definitely related,” as appropriate.
- **Unlikely to be related** – A clinical event, including an abnormal laboratory test result, whose temporal relationship to drug administration makes a causal relationship improbable (e.g., the event did not occur within a reasonable time after administration of the trial medication) and in which other drugs or chemicals or underlying disease provides plausible explanations (e.g., the participant’s clinical condition, other concomitant treatments).
- **Not Related** – The AE is completely independent of study drug administration, and/or evidence exists that the event is definitely related to another etiology. There must be an alternative, definitive etiology documented by the clinician.
  - 1. EXPECTEDNESS

The PIs will be responsible for determining whether an AE is expected or unexpected. An AE will be considered unexpected if the nature, severity, or frequency of the event is not consistent with the risk information previously described for the study agent.

8.2 TIME PERIOD AND FREQUENCY FOR EVENT ASSESSMENT AND FOLLOW-UP

The occurrence of an AE or serious adverse event (SAE) may come to the attention of study personnel during study visits and interviews of a study participant presenting for medical care, or upon review by a study monitor.

All AEs including local and systemic reactions not meeting the criteria for SAEs will be captured on the appropriate case report form. Information to be collected includes event description, time of onset, clinician’s assessment of severity, relationship to study product (assessed only by those with the training and authority to make a diagnosis), and time of resolution/stabilization of the event.

All AEs occurring while on study must be documented appropriately regardless of relationship. All AEs will be followed to adequate resolution.

Changes in the severity of an AE will be documented to allow an assessment of the duration of the event at each level of severity to be performed. AEs characterized as intermittent require documentation of onset and duration of each episode.

The PI will record all reportable events with start dates occurring any time after informed consent is obtained until 7 days after the administration of investigational medication. At each study visit, the investigator will inquire about the occurrence of AE/SAEs since the last visit. Events will be followed for outcome information until resolution or stabilization.

- 1. REPORTING PROCEDURES
     1. ADVERSE EVENT REPORTING

Adverse events will be reported to the IRB on an annual basis and to the Sponsor at designated Safety Review milestones as in Section 6.1.7.

- - 1. SERIOUS ADVERSE EVENT REPORTING

The investigators will report all adverse events to the Sponsor and local IRB. Serious, unexpected adverse events will be reported to the local IRB and to the Sponsor within 5 business days. All other AEs (unexpected but not serious or expected) will be reported to the Sponsor as a summary at the completion of each group of 6 patients receiving a specific dose, and at the end of the study. All other AEs will be reported to the IRB at the time of regular (i.e. yearly) continuing review submissions.

The Sponsor will report all serious, unexpected adverse events as an IND safety report to the FDA no later than 15 calendar days after the sponsor’s initial receipt of this information. Fatal or life- threatening unexpected experiences for which there is a possibility that the experience may have been caused by the drug will be reported by the Sponsor to the FDA by telephone or facsimile transmission no later than 7 calendar days after receipt of this information. An annual report which includes summaries of all IND safety reports will be submitted to the FDA each year in the annual report by the Sponsor.

All SAEs will be followed until satisfactory resolution or until the site investigator deems the event to be chronic. Other supporting documentation of the event may be requested by the DCC/study sponsor and should be provided as soon as possible.

The study Sponsor will be responsible for notifying FDA of any unexpected fatal or life- threatening suspected adverse reaction as soon as possible but in no case later than 7 calendar days after the Sponsor's initial receipt of the information.

- - 1. UNANTICIPATED PROBLEM REPORTING

Incidents or events that meet the OHRP criteria for UPs require the creation and completion of an UP report form. It is the Site Investigator’s responsibility to report UPs to their IRB and to the Study Sponsor.

To satisfy the requirement for prompt reporting, UPs will be reported using the following timeline:

- - - - UPs will be reported to the IRB and to the study Sponsor within 5 business days of the Investigator becoming aware of the problem.
    1. EVENTS OF SPECIAL INTEREST Not applicable.
    2. REPORTING OF PREGNANCY

In the event that a subject becomes pregnant after screening and before enrollment, that subject will be excluded from participation. Pregnancy that occurs after receipt of investigational medicine will prompt reporting to the patient’s primary physician for appropriate monitoring and follow-up. All pregnancies will be followed by the study team through outcome and reported to the appropriate regulatory agencies.

- 1. STUDY HALTING RULES

Administration of study agent will be halted under the circumstances described below. The Principal Investigators will halt enrollment immediately and notify the Sponsor within 24 hours. The Study Sponsor will inform the FDA of the temporary halt and the disposition of the study.

Enrollment in the study will be terminated if the any of following occur:

- One Grade 4 toxicity**1**, regardless of relation to investigational medicine (toxicity grades based on NCI CTCAE v4.03)(6)**10**
- One Grade 3**2** or higher toxicity of any type, if determined to be probably or definitely related to the investigational medication in a patient who was randomized to SP16 or is unexpected in nature.

Enrollment in the study will be halted until joint review by the PIs and Sponsor if any of the following occur:

- Two identical toxicities of Grade 1 or higher in two separate subjects
- One thrombotic event of Grade 1 or higher
- One hemorrhage of Grade 1 or higher
- One blood/bone marrow event of Grade 2 or higher

In addition, the Principal Investigators may choose to halt enrollment, if in their opinion, continued

1 Life-threatening; urgent intervention indicated

2 Severe; or medically significant but not immediately life- threatening; hospitalization or prolongation of hospitalization indicated; disabling

enrollment would pose an unacceptable risk to subjects. Additional description of halting and stopping is provided in Section 5.5.

- 1. SAFETY OVERSIGHT

Safety oversight will be under the direction of the Principal Investigators and Sponsor. The Principal Investigators and Sponsor will review safety data after the first three subjects within a dosing level have completed follow-up and again after all subjects within a dosing level have completed follow-up. In addition, the occurrence of any adverse events specified in Section 8.5 will prompt a safety review of the Principal Investigators and Sponsor.

1. CLINICAL MONITORING

Site monitoring is conducted to ensure that the rights and well-being of human subjects are protected, that the reported trial data are accurate, complete, and verifiable, and that the conduct of the trial is in compliance with the currently approved protocol/amendment(s), with GCP, and with applicable regulatory requirement(s).

- Monitoring for this study will be performed by the Principal Investigators.
- Complete review of Case Report Forms will be performed by the Principal Investigators during Safety Milestones as described in Section 6.1.7.

1. STATISTICAL CONSIDERATIONS
   1. ANALYSIS DATASETS
      - All analyses will be conducted on the Safety Analysis Dataset (e.g., participants who took at least one dose of investigational product).
   2. DESCRIPTION OF STATISTICAL METHODS

10.2.1 GENERAL APPROACH

Baseline measurements and demographic characteristics will be summarized with median and interquartile range. Descriptive summaries of categorical measurements will consist of frequencies and proportions. The summaries for each measurement will be provided separately for each treatment group (i.e., 0.0125 mg/kg group, 0.050 mg/kg group, 0.200 mg/kg group, placebo group). Between group comparisons across all four treatment groups will be conducted with the chi-square test for categorical variables and the Kruskal-Wallis test for continuous variables. An additional analysis will compare the pooled active SP16 groups against the placebo group using the chi-square test for categorical variables and the Mann-Whitney U test for continuous variables. An assessment of sample size or power analyses is not performed for this Phase I study. Formal sample sizes were not calculated based on statistical considerations. The sample sizes specified are based on standard practice in early Phase 1 studies. A two-sided p-value < 0.05 will be considered statistically significant.

- 1. MEASURES TO MINIMIZE BIAS
     1. ENROLLMENT / RANDOMIZATION / MASKING PROCEDURES Subjects will be randomized in a 3:1 ratio to either active SP16 or matching placebo.

Randomization will occur such that at least 1 of the first 3 subjects within a dosing level will receive active SP16.

The VCU Investigational Drug Pharmacy will be responsible for blinding. Blinding will be accomplished through the use of syringes of indistinguishable appearance.

- - 1. BREAKING THE STUDY BLIND/PARTICIPANT CODE

Allocation will remain blinded to subjects and study investigators until necessary for Safety Milestone assessments described above. In addition, unblinding may occur if requested by a licensed physician in order to treat an AE that occurs during the study period.

1. SOURCE DOCUMENTS AND ACCESS TO SOURCE DATA/DOCUMENTS

Virginia Commonwealth University will maintain appropriate medical and research records for this trial, in compliance with ICH E6 and regulatory and institutional requirements for the protection of confidentiality of participants.

1. QUALITY ASSURANCE AND QUALITY CONTROL

The PIs will oversee all data collection and recording on Case Report Forms. They will periodically review Case Report Forms as described above in Section 9.

1. ETHICS/PROTECTION OF HUMAN SUBJECTS
   1. ETHICAL STANDARD

Principal Investigators will ensure that this study is conducted in full conformity with Regulations for the Protection of Human Subjects of Research codified in 45 CFR Part 46, 21 CFR Part 50, 21 CFR Part 56, and/or the ICH E6.

- 1. INSTITUTIONAL REVIEW BOARD

The protocol, informed consent form(s), recruitment materials, and all participant materials will be submitted to the IRB for review and approval. Approval of both the protocol and the consent form must be obtained before any participant is enrolled. Any amendment to the protocol will require review and approval by the IRB before the changes are implemented to the study. All changes to the consent form will be IRB approved; a determination will be made regarding whether previously consented participants need to be re-consented.

- 1. INFORMATED CONSENT PROCESS
     1. CONSENT/ASSENT AND OTHER INFORMATIONAL DOCUMENTS PROVIDED TO PARTICIPANTS

Consent forms describing in detail the study agent, study procedures, and risks are given to the participant and written documentation of informed consent is required prior to starting intervention/administering study product.

- - 1. CONSENT PROCEDURES AND DOCUMENTATION

Informed consent is an ongoing process that is initiated prior to the individual’s agreeing to participate in the study and continues throughout the individual’s study participation. Extensive discussion of risks and possible benefits of participation will be provided to the participants and their families. Consent forms will be IRB-approved and the participant will be asked to read and review the document. The investigator will explain the research study to the participant and answer any questions that may arise. All participants will receive a verbal explanation in terms suited to their comprehension of the purposes, procedures, and potential risks of the study and of their rights as research participants. Participants will have the opportunity to carefully review the written consent form and ask questions prior to signing.

The participants should have the opportunity to discuss the study with their surrogates or think about it prior to agreeing to participate. The participant will sign the informed consent document

prior to any procedures being done specifically for the study. The participants may withdraw consent at any time throughout the course of the trial. A copy of the informed consent document will be given to the participants for their records.

- 1. PARTICIPANT AND DATA CONFIDENTIALITY

Participant confidentiality is strictly held in trust by the participating investigators, their staff, and the sponsor(s) and their agents. This confidentiality is extended to cover testing of biological samples in addition to the clinical information relating to participants. Therefore, the study protocol, documentation, data, and all other information generated will be held in strict confidence.

- Coded identifiers will be attached to data or samples.
- The key to coded identifiers will be stored in a password-protected database compliant with local standards and will be accessible only to the research team. Case Report Forms and written informed consent forms will be stored in a locked cabinet within a locked office with access restricted to the study team.
- Coded data and samples, but not the key, will be shared with the Sponsor.

The study participant’s contact information will be securely stored at the clinical site for internal use during the study. At the end of the study, all records will continue to be kept in a secure location for as long a period as dictated by local IRB and Institutional regulations.

- - 1. RESEARCH USE OF STORED HUMAN SAMPLES, SPECIMENS OR DATA
       - Intended Use: Samples and data collected under this protocol may be used to accomplish the objective of this study. No genetic testing will be performed.
       - Storage: Access to stored samples will be limited using physical restrictions to locked storage areas and coded identifiers. Samples and data will be stored using codes assigned by the investigators. Data will be kept in password- protected computers. Only investigators will have access to the samples and data.
  1. FUTURE USE OF STORED SPECIMENS Not applicable.

1. DATA HANDLING AND RECORD KEEPING
   1. DATA COLLECTION AND MANAGEMENT RESPONSIBILITIES

Data collection is the responsibility of the clinical trial staff at the site under the supervision of the site PI. The investigator is responsible for ensuring the accuracy, completeness, legibility, and timeliness of the data reported.

All source documents should be completed in a neat, legible manner to ensure accurate interpretation of data. Black ink is required to ensure clarity of reproduced copies. When making changes or corrections, cross out the original entry with a single line, and initial and date the change. Clinical data (including AEs, concomitant medications, and expected adverse reactions data) and clinical laboratory data will be entered onto Case Report Forms.

- 1. STUDY RECORDS RETENTION

Study documents should be retained for a minimum of 5 years beyond completion of this study or 2 years after the last approval of a marketing application in an ICH region and until there are no pending or contemplated marketing applications in an ICH region or until at least 2 years have elapsed since the formal discontinuation of clinical development of the investigational product, whichever is later. No records will be destroyed without the written consent of the Sponsor, if

applicable. It is the responsibility of the Sponsor to inform the investigator when these documents no longer need to be retained.

- 1. PROTOCOL DEVIATIONS

A protocol deviation is any noncompliance with the clinical trial protocol, GCP, or MOP requirements. The noncompliance may be either on the part of the participant, the investigator, or the study site staff. As a result of deviations, corrective actions are to be developed by the site and implemented promptly.

These practices are consistent with ICH S7A, B:**11,12**

- - - 4.5 Compliance with Protocol, sections 4.5.1, 4.5.2, and 4.5.3
    - 5.1 Quality Assurance and Quality Control, section 5.1.1
    - 5.20 Noncompliance, sections 5.20.1, and 5.20.2.

It is the responsibility of the site to use continuous vigilance to identify and report deviations within 3 working days of identification of the protocol deviation. All deviations must be addressed in study source documents, reported to the local IRB per their guidelines. The site PI/study staff is responsible for knowing and adhering to their IRB requirements.

- 1. PUBLICATION AND DATA SHARING POLICY

The results of this study will not be published in a peer-reviewed medical journal. This study will not be registered on clinicaltrials.gov.

1. STUDY ADMINISTRATION
   1. STUDY LEADERSHIP

The PIs and Sponsor will serve as study leadership.

1. CONFLICT OF INTEREST POLICY

The independence of this study from any actual or perceived influence, such as by the pharmaceutical industry, is critical. Therefore, any actual conflict of interest of persons who have a role in the design, conduct, analysis, publication, or any aspect of this trial will be disclosed and managed. Furthermore, persons who have a perceived conflict of interest will be required to have such conflicts managed in a way that is appropriate to their participation in the trial.

1. APPENDIX

| **Version** | **Date** | **Significant Revisions** |
| --- | --- | --- |
| v.0.1 | 14 December 2017 | Not applicable |
| v.2 | 27 April 2018 | 1. Recommendations from FDA review (ECG to 30 minutes post-dose); 2. Addition of +/- times for study procedures; 3. Shortening of Visit 1 to 12 hours direct observation, followed by 24 hour in person visit; 4. 24 hours assessment split into separate visit; 5. Clarification of placebo produced by IDS Pharmacy at VCU. |
| V. 3 | 10 July 2018 | 1. Update protocol version and date 2. Added “all pregnancies will be followed by the study team through outcome and reported to the   appropriate regulatory agencies.” To section 8.4.5   1. Deleted DSMB language (3 sentences) from section   8.5 as recommended by FDA |
|  |  |  |
|  |  |  |
|  |  |  |
|  |  |  |
|  |  |  |
|  |  |  |
|  |  |  |
|  |  |  |
|  |  |  |
|  |  |  |
|  |  |  |
|  |  |  |

1. LITERATURE REFERENCES

1 Gooptu B, Lomas DA. Conformational pathology of the serpins: themes, variations, and therapeutic strategies. Annu Rev Biochem. 2009 Jan;78:147–76.

2 Strickland DK, Muratoglu SC, Antalis TM. Serpin-Enzyme Receptors LDL Receptor-Related Protein 1. Methods Enzymol. 2011;499:17–31.

3 Perlmutter DH, Joslin G, Nelson P, Schasteen C, Adams SP, Fallon RJ. Endocytosis and degradation of alpha 1-antitrypsin-protease complexes is mediated by the serpin-enzyme complex (SEC) receptor. J Biol Chem. 1990 Oct 5;265(28):16713–6.

4 Stefano Toldo, Dana Austin, Adolfo G. Mauro, Eleonora Mezzaroma, Benjamin W. Van Tassell, Carlo Marchetti, Salvatore Carbone, Soren Mogelsvang, Cohava Gelber, Antonio Abbate. Low- Density Lipoprotein Receptor–Related Protein-1 Is a Therapeutic Target in Acute Myocardial Infarction. J AAC : basic to translational science vol . 2 , n o . 5 , 2017

5 Mozaffarian D, Benjamin EJ, Go AS, et al. Heart disease and stroke statistics — 2016 update. A report from the American Heart Association. Circulation. 2016 Jan 26;133(4):e38-360.

6 Roubille F, Samri A, Cornillet L, et al. Routinely-feasible multiple biomarkers score to predict prognosis after revascularized STEMI. Eur J Intern Med. 2010 Apr;21(2):131–6.

7 Office of Training and Communications. Guidance for Industry: Estimating the Maximum Safe Starting Dose in Initial Clinical Trials for Therapeutics in Adult Healthy Volunteers. Rockville, MD: 2005.

8 Abbate A, Van Tassell BW, Christopher S, et al. Effects of Prolastin C (Plasma-Derived Alpha-1 Antitrypsin) on the acute inflammatory response in patients with ST-segment elevation myocardial infarction (from the VCU-alpha 1-RT pilot study). Am J Cardiol 2015;115(1):8–12.

9 Office of Training and Communications. Guidance for Industry: E14 Clinical Evaluation of QT/QTc Interval Prolongation and Proarrhythmic Potential for Non-Antiarrhythmic Drugs. Rockville, MD: 2005.

10 National Institutes of Health. National Cancer Institute Common Terminology Criteria for Adverse Events (CTCAE) version 4.03. Available from: https://evs.nci.nih.gov/ftp1/CTCAE/CTCAE_4.03_2010-06-14_QuickReference_5x7.pdf.

11 ICHS7A-

<http://www.ich.org/fileadmin/Public_Web_Site/ICH_Products/Guidelines/Safety/S7A/Step4/S7A_G> uideline.pdf

12 ICHS7b-

[http://www.ich.org/fileadmin/Public_Web_Site/ICH_Products/Guidelines/Safety/S7B/Step4/S7B_G](http://www.ich.org/fileadmin/Public_Web_Site/ICH_Products/Guidelines/Safety/S7B/Step4/S7B_Guideline.pdf) [uideline.pdf](http://www.ich.org/fileadmin/Public_Web_Site/ICH_Products/Guidelines/Safety/S7B/Step4/S7B_Guideline.pdf)
